# Reinventory of the vascular plants of Mormon Island Crane Meadows after forty years of restoration, invasion, and climate change

**DOI:** 10.1016/j.heliyon.2022.e09640

**Published:** 2022-06-03

**Authors:** A.J. Caven, J.D. Wiese

**Affiliations:** Platte River Whooping Crane Maintenance Trust, Wood River, NE 68883, USA

**Keywords:** Lowland tallgrass prairie, Long-term research, Prairie restoration, Invasive species, Central platte river valley, Floristic quality index, Biological homogenization, Remnant prairies, Habitat fragmentation, Great plains

## Abstract

The majority of tallgrass prairie has been lost from North America's Great Plains, but remaining tracts often support significant biodiversity. Despite permanent protections for some remnants, they continue to face anthropogenic threats including habitat fragmentation, invasive species, and climate change. Conservationists have sought to buffer remnants from threats using prairie restoration but limited research has assessed such practices at the landscape-level. We reexamine the flora of Mormon Island, the largest tract of lowland tallgrass prairie remaining in the Central Platte River Valley (CPRV) of Nebraska, USA, nearly 40-years after it was initially inventoried and following widespread restoration. We also conducted preliminary inventories of nearby Shoemaker Island and adjacent off-island habitats using an ecotope-based stratified random sampling approach. We examined change at Mormon Island between 1980-1981 and 2015–2020 and compared it to adjacent conservation lands using a number of vegetation indices. We documented 389 vascular plant species on Mormon Island, 405 on Shoemaker Island, and 337 on off-island habitats from 2015-2020, which represented an increase in native and exotic species richness on Mormon Island compared to 1980–1981 results. Floristic quality index (FQI) values increased at Mormon Island between 1980-1981 and 2015–2020. Paradoxically, the distribution of exotic-invasive species also expanded. Mormon Island from 2015-2020 was more similar to Shoemaker Island and off-island habitats from 2015-2020 than Mormon Island from 1980-1981. Widespread restoration introduced a number of high conservation value species native to Nebraska but novel to the CPRV, which improved FQIs despite increased exotic species invasion. These concurrent trends appear to have driven biological homogenization across the study area. Restoration did not fully buffer Mormon Island from exotic species invasion but it may have partially mitigated the impact considering the persistence of most native species across a 40-year period. We recommend using “local ecotype” seed for restorations to preserve distinctive local communities.

## 1 Introduction

The Great Plains of North America is one of the most transformed landscapes in the world (Dahl, 2000, Samson et al., 2004; Engle et al., 2008; Wright and Wimberly 2013). However, where remnant tracts of grassland and wetland exist, they often support high levels of biodiversity and distinct ecological communities (Polley et al., 2005; Hendrix et al., 2010; Caven et al., 2017). Protecting and enhancing remnant sites is essential as restoration techniques to date have generally failed to replicate the functional traits and diversity of relict systems (Polley et al., 2005; Aronson and Galatowitsch 2008; Newbold et al., 2020). Widespread efforts have been made to protect and enhance important remnant sites, but they continue to be impacted directly and indirectly by a number of anthropogenic drivers (Nelson et al., 2006; Hobbs et al., 2009; Alstad et al., 2016). For instance, the restoration of adjacent crop fields to native grassland can buffer remnant sites from exotic-invasive species colonization (Rowe, 2013), but can itself introduce novel species into a region when local ecotype seed is not used (Larson et al., 2021). Concurrently, biological invasions are an increasing problem and can lead to regime shifts in local and regional floras (Sharma et al., 2005; Seabloom et al., 2006; Hobbs et al., 2009). Species community composition and distributions have also shifted in response to climate change, human disturbance patterns, and continued habitat fragmentation (Seabloom et al., 2006; Austin and Van Niel 2011; Wilson et al., 2016). Alstad et al. (2016) suggests that annual rates of species colonization and extinction are increasing in remnant tallgrass prairies. Considering the myriad of influences on remnant systems, it is essential to track species composition periodically and over long durations in order to assess ecosystem conditions and adjust management practices in accordance with emerging problems (Aronson and Galatowitsch 2008; Alstad et al., 2016).

Riparian prairie and embedded wetlands in the Central Platte River Valley (CPRV) of Nebraska have largely been protected and restored because they represent important habitat components for migratory Whooping Cranes (*Grus americana*) regionally (USFWS 1978; USFWS 1981; Currier et al., 1985, Baasch et al., 2019). However, this biologically important and unique ecosystem, with its distinctive hydrology, hosts significant plant diversity on which a variety of wildlife depends (Nagel and Kolstad 1987; Currier 1989; Currier and Henszey 1996; Henszey et al., 2004; Rolfsmeier and Steinauer 2010; Kaul et al., 2012; Brown and Johnsgard 2013; Caven et al., 2017; Brinley Buckley et al., 2021a; 2021b). Nagel and Kolstad (1987) documented the vascular plant communities of two remnant conservation proprieties in the CPRV in the early 1980s (1980–1981), the Crane Trust (i.e., Mormon Island Crane Meadows) and Audubon's Rowe Sanctuary, but little long-term vegetation research has been published from this region in recent decades (Johnson 1997; Henszey et al., 2004). Several techniques for restoring riparian wetlands and grasslands were developed in the CPRV over the last 40 years and restoration efforts continue today (Pfeiffer 1999; Whitney 1999; Meyer et al., 2010). However, their impact on the present floral community remains understudied and limited to relatively small-scale post restoration assessments (Meyer et al. 2008, 2010).

Like the Great Plains at large, the CPRV has been radically transformed by a number of anthropogenic forces since European settlement (Williams 1978; Sidle et al., 1989; Dahl, 2000; Samson et al., 2004). Lowland prairie and wetland landcover have been significantly reduced as a result of agricultural expansion, as this biologically important area is also one of the most productive agricultural regions in the Great Plains (Currier et al., 1985, Sidle et al., 1989; Dappen et al., 2008). Furthermore, the hydrology of the Platte River has been profoundly altered by damming, diversion (e.g., canals), and groundwater pumping, resulting in large decreases in annual discharge, peak flows, river width, and groundwater levels that maintain riparian wetlands (Currier 1989; Wesche et al., 1994; Simons and Associates 2000; Henszey et al., 2004; Murphy et al., 2006; Caven et al., 2019a). Additionally, the removal of natural and periodic disturbances from the river valley, such as significant flood pulses and wildfires, resulted in large increases in riparian woodland and shrubland landcover manifest as accretion within the former channel bed as well as encroachment into remaining herbaceous habitats (Williams 1978; Currier 1982; Caven et al., 2019b; Fogarty et al., 2020). Furthermore, the disappearance of historic grazers and browsers such as Plains *Bison* (*Bison bison*) and Elk (*Cervus canadensis*), as well as their concurrent replacement by domesticated Cattle (*Bos taurus*), also likely altered patterns of vegetation composition in remaining herbaceous habitats (Hart 2001; Towne et al., 2005; Fricke et al., 2008; O'Connor et al., 2020). However, beginning in the mid-1970s private conservation organizations began to protect, restore, manage, and study herbaceous habitats in the CPRV for the benefit of migratory birds, particularly the Whooping Crane, as this area was designated “critical habitat” for the species under the Endangered Species Act in 1978 (USFWS 1978, Currier et al., 1985, Pfeiffer 1999, Vrtiska and Sullivan, 2009; Caven et al., 2019b, 2020).

**Figure 1:**
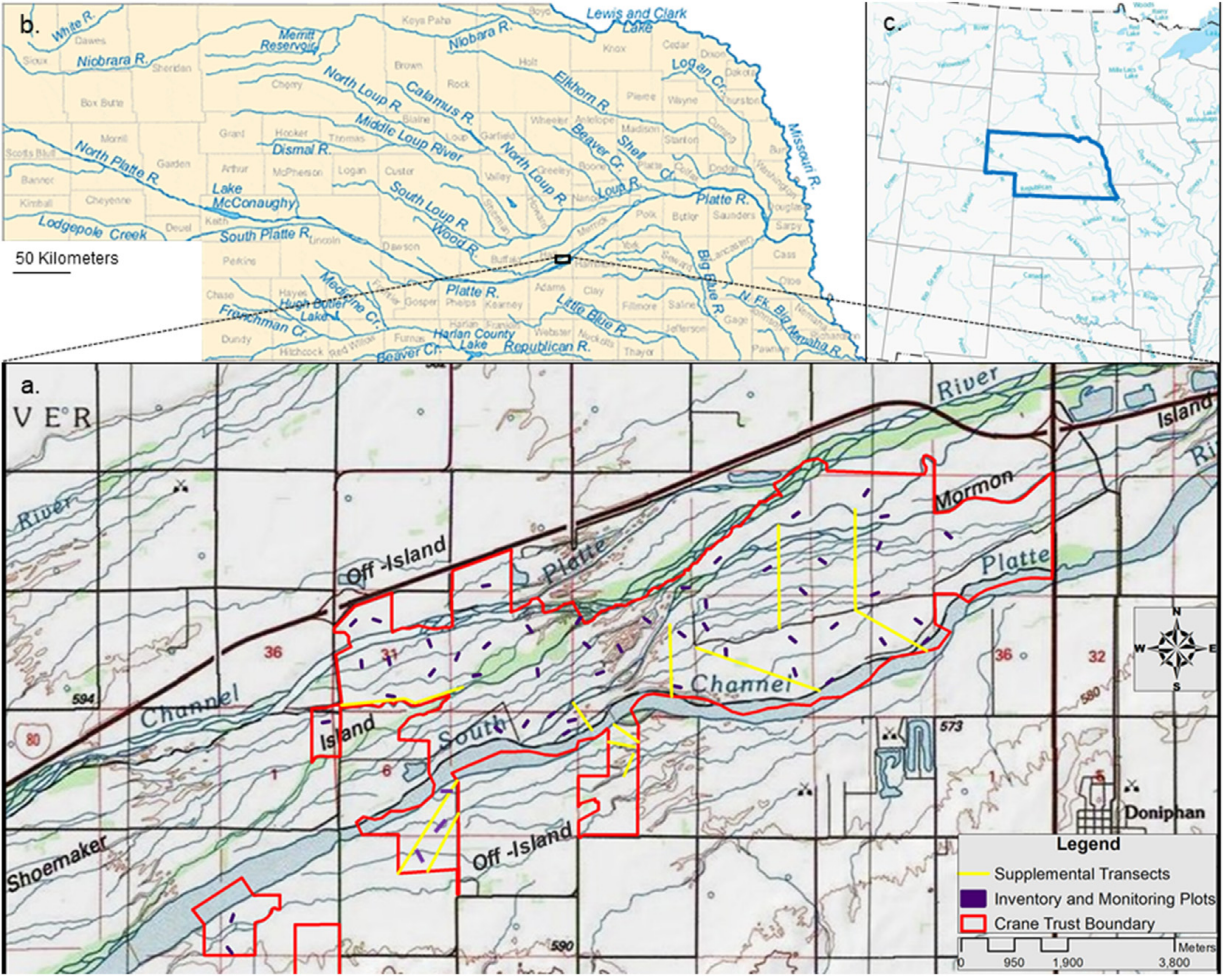
Core study area map (a) including vegetation inventory and monitoring plots (purple), supplemental transects (yellow), and the Crane Trust's main complex property boundary (red), along with labeled depictions of the South (main) Channel of the Platte River, Mormon Island, Shoemaker Island, off-island habitats, and the village of Doniphan, Nebraska. The graphic also includes additional maps highlighting the core study area's location within the state of Nebraska and along the Platte River (b; black rectangle) as well as Nebraska's location within the United States portion of the Great Plains of North America (c; blue polygon). Two off-island sites (Dippel Tract) are not pictured in graphic “a” above and exist about 24 km west of the Crane Trust's main complex (40.706397°, -98.788919°; 620 m elevation). Map created in Arc Map 10.8.2, Esri, Redlands, CA, USA. Imagery from National Geographic Society, Washington, D.C., USA, and i-cubed, Fort Collins, CO, USA.

Concurrent with 40 years of restoration work, research has generally documented an increase in herbaceous landcover in areas of the CPRV with high conservation ownership (Pfeiffer 1999; Krapu et al., 2014; Caven et al., 2019b). However, little research has documented long-term changes in the floral composition of these protected and restored landscapes since conservation efforts began (Pfeiffer 1999; Meyer et al., 2010). Our primary objective was to conducted a reinventory of the vascular plants of Mormon Island, which was originally assessed by Nagel and Kolstad (1987) from 1980-1981, to investigate shifts in the vascular plant community over time. Our secondary objective was to developed an initial inventory of the flora of Shoemaker Island and adjacent non-island (i.e., bank) habitats owned and managed by the Crane Trust to examine variation in the vegetation community across spatially distinct yet proximate components of the CPRV and implement a framework for future long-term monitoring (Figure 1). Finally, we explored potential explanations for patterns of species composition and shifts therein over time including restoration practices, invasive species, climate change, and hydrology.

## 2 Methods

### 2.1 Study site

The Platte River is a braided river that includes a number of small, mobile, and temporary islands (i.e., sandbars) that support limited early successional vegetation (Smith 1971; Currier 1982; Simon and Associates 2000). However, this riverine ecosystem also supports a number of significant anabranches around permanently stabilized and vegetated islands (Currier 1982; Horn et al., 2012; Caven et al., 2019b). Some stabilized islands were formed more recently as the result of significant decreases in flow and sediment transportation associated with damming and diversion following European settlement and these islands tend to support early successional woodland communities (Williams,^1978^; Currier 1982; Johnson 1997; Caven et al., 2019b). However, relatively substantial anabranches encircle the oldest and largest islands within this braided river floodplain and support the most distinctive and developed herbaceous vegetation communities (Currier 1982; Nagel and Kolstad 1987; Currier and Davis 2000; Henszey et al., 2004; Rolfsmeier and Steinauer 2010). These larger permanent islands, including Mormon and Shoemaker Islands, as well as the adjacent landscape comprise the focal area for this study (Figure 1). The eastern edge of our core study area is defined by where U.S. Highway 281 bisects Mormon Island about 13 km southwest of Grand Island, Nebraska (40.8110°, -98.3788°; 574 m elevation; Figure 1). The western edge of our core study area borders the south channel of the Platte River about 10 km southeast of Wood River, Nebraska (40.760071°, -98.514464°; 590 m elevation; Figure 1). Additionally, we surveyed two off-island sites (Dippel Tract) about 24 km west (40.706397°, -98.788919°; 620 m elevation) of our core study area approximately 7 km southeast of Gibbon, Nebraska.

The tallgrass prairie extends west of its general ecoregion within the Platte River Valley as a result of subirrigation via shallow groundwater supported by the river (Currier 1989; Rolfsmeier and Steinauer 2010; Kaul et al., 2012). Lowland tallgrass prairie predominates in many conservation lands in the CPRV but seasonal wetlands such as wet meadows and semipermanent wetlands such as shallow marshes prevail in areas of lower topography and elevation relative to the channel bed (Currier 1989; Henszey et al., 2004; McKee 2006; Kaul et al., 2012; Rolfsmeier and Steinauer 2013). The river valley includes a sizeable hyporheic zone and relatively shallow soils, generally ranging from fine sand to loam with varying degrees of organic matter, present atop a deep layer of coarse alluvium (Chen et al., 2007; Dillion et al., 2016; USDA 2004, 2009; 2021b). The river valley as a whole represents a highly connected hydrological system where groundwater elevation and wetland inundation are largely tied to fluctuations in river discharge (Wesche et al., 1994; Chen et al., 2007; Brinley Buckley et al., 2021a). There is also extensive riparian woodland bordering the Platte River, particularly in former and now dry channel beds (Williams 1978; Currier, 1982; Johnson 1997; Caven et al., 2019b). Large-scale efforts to remove riparian woodland from near the main channel of the Platte River have been undertaken to promote herbaceous habitat and widen channel areas for Whooping Crane and Sandhill Crane use (Currier, 1991; McKee 2006; Caven et al., 2019b). Furthermore, significant expanses of former crop field have been returned to grassland via restoration with “native” seed mixes of varying species richness (Pfeiffer 1999; Whitney 1999). Earlier restorations in the CPRV predominantly included a standard tallgrass prairie seed mix while later restorations increasingly included a wider variety of local ecotype seed and landscape recontouring to reflect historic topography where land had been leveled for agriculture (Pfeiffer 1999; Meyer et al., 2010). Early efforts to restore natural herbaceous communities within the footprints of cleared riparian forests simply involved post-hoc management to limit woody recolonization (e.g., controlled burns, mechanical control, etc.). However, after limited success establishing appropriate vegetation communities through natural colonization, land managers began inter-seeding restorations within former riparian woodlands (Currier 1994; Pfeiffer 1999). Herbaceous habitats were managed with controlled burning and rotational grazing to promote native biodiversity and landscape heterogeneity (Crane Trust 1998; Fuhlendorf et al., 2009) and most exotic-invasive species present were regularly managed with best practices for individual species control (e.g., selective herbicides, mowing, etc.; Crane Trust 1998; Galatowitsch et al., 2016).

### 2.2 Sampling design

The vegetation inventory and monitoring (I&M) plot system was designed based on an “ecotope” concept in which polygons delineated distinct combinations of soil type (e.g., Platte-Bolent complex), flooding frequency (e.g., occasionally flooded), land use history (e.g., relict), and vegetation community type (e.g., herbaceous) (Naveh 1994; Herrick et al., 2009; Caven et al., 2017). An “ecotope” is the smallest unit of analyses in landscape ecology that is clearly mappable, relatively homogenous, and based on spatially explicit biotic (e.g., vegetation) and abiotic (e.g., hydrology) ecosystem components (Klijn and Udo de Haes 1994; Naveh 1994; Ingegnoli 2013). Soil type and flooding frequency data was derived from the USDA–NRCS (2021b), while land use history and vegetation community type were derived from internal organizational records (Caven et al., 2017), previous reports and publications (Nagel 1981; Henszey et al., 2004), and aerial imagery (USBR 1938; Google 2015). We took a stratified random sampling approach that included sufficient replication of distinct ecotopes to allow for the assessment of land management impacts on the vegetation community while also providing a robust inventory (Naveh 1994; Elzinga et al., 2009; Herrick et al., 2009; Caven et al., 2017). This process yielded 59 I&M plots distributed across 5,723 ac (2316 ha) and we surveyed an additional 640 ac (259 ha) using 12 supplemental walking transects. In total, we installed one I&M plot for every 97 ac (39 ha). Twenty-three I&M plots were distributed across Mormon Island, 26 across Shoemaker Island, and 10 across off-island sites in accordance with parcel sizes and landscape complexity (i.e., variation in soil type, land use history, and flooding frequency). In total, our sampling efforts covered 6,363 ac (2,575 ha; Figure 1).

### 2.3 Survey methods

Field survey methods were largely based on Symstad et al. (2008) and are described in detail in Caven et al. (2017). We used two complementary vegetation survey methods including the point-line intercept method, which provides dominant species and habitat structure information, and the quadrat ocular cover estimation method, which provides species diversity and richness information (Symstad et al., 2008; SODN 2012; Herrick et al., 2009). Ground cover and the dominant species within 3 height classes (<0.5 m, 0.5 ≤ 2.0 m, >2.0 m) were recorded every 2.0 m along permanently marked 100-m transects (Herrick et al., 2009; SODN 2012; Caven et al., 2017). Additionally, species richness and relative abundance were recorded every 10 m along the same transect using a 0.5 × 1.0 m quadrat marked every 10 cm along the frame to facilitate cover estimation (Symstad et al., 2008; Caven et al., 2017). Percent cover was valued to the nearest 5% allowing for overlapping species canopies; therefore, total cover estimates regularly exceeded 100% per plot (Daubenmire 1959; Symstad et al., 2008; Caven et al., 2017). We additionally visually surveyed a 200 m buffer centered at the monitoring plot starting point (meter 0) for the presence of species not detected via point-line intercept or quadrat surveys. Data was collected at each of the 59 I&M plots at least every other year from 2015 to 2019 resulting in a total of 170 surveys completed. Survey data was collected during the frost-free growing season when plants were sufficiently mature to identify between 15 June and 16 October, but most sites were visited between the last week of June and the first week of September annually.

In June of 2018 and 2019 we noted the presence of all species visually detected along supplemental walking transects, which varied in length depending on landscape features from 250 m to 1,610 m. Six of the 12 transects represented those surveyed during the initial inventory of Mormon Island (See Kolstad 1981; Nagel 1981; Nagel and Kolstad 1987), while the others were located within areas of Shoemaker Island and off-island habitats not included in the I&M plot framework (Figure 1). We also noted novel species encountered while walking to and from I&M plots. Species documented while walking supplemental transects or *en route* to and from I&M plots were considered “incidental” detections (Table 1). Our inventory also includes novel species incidentally detected during field work in 2020. Incidental detections improve the comprehensiveness of species lists and allow us to estimate the effectiveness of our I&M system (i.e., percent detected via the I&M system vs “incidentally” observed; Watson 2004; Chen et al., 2013; Guralnick et al., 2018). We also produced a species accumulation curved based on the number of plots surveyed following the sample-based rarefaction method proposed by Chiarucci et al. (2008) using the “vegan” package in the open-source statistical software program R (Oksanen et al., 2020; R Core Team 2020).

**Table 1:** Vascular plant species detected on land owned or managed by the Platte River Whooping Crane Maintenance Trust including results from surveys conducted by Nagel and Kolstad (1987) from 1980 to 1981 at Mormon Island as well as from 2015 to 2020 at Mormon Island, Shoemaker Island, and at non-island habitats along the north and south banks of the Platte River (Off-Island). Presence on Mormon Island in 1980–1981 per Nagel and Kolstad (1987) is indicated by an “X”, for 2015–2020 surveys results are given as the percent of monitoring plots at which each species was detected per location category. For species not detected at monitoring plots but encountered incidentally, presence per location category is indicated by an “i”. For species detected in the years between Nagel and Kolstad (1987) and recent surveys (2015–2020) via occasional floral assessments the year of the herbarium record is given. Results are presented along with whether the species represents a new record (Coun. Record) per Kaul et al. (2012) in Hall or Buffalo Counties, species Wetland Indicator Status (WIS; Lichvar, 2014) as obligate wetland (OBL), facultative wetland (FACW), facultative (FAC), facultative upland (FACU), or upland (UPL), status as native (N), exotic (E), or both (B) via USDA-NRCS (2021a), conservation status (Conserv. Status) as Tier-1 (T1) or Tier-2 (T2) under the Nebraska Natural Legacy Program (Schneider et al., 2018), growth habitat as tree, forb, shrub, vine, or graminoid (Gram.) also per USDA-NRCS (2021a), and coefficient of conservatism (0–10) per the Nebraska Natural Heritage Program state vascular plant list (Rolfsmeier and Steinauer 2013) with low values representing species that are common and can tolerate a wide range of ecological conditions including unnatural disturbances and high values representing rare plants requiring a relatively narrow range of conditions.

Family	*Genus species* Authority	Coun. Record	Nagel and Kolstad (1987)	^†^Mormon 2015–2020	^‡^Shoemaker 2015–2020	∗Off-Island 2015–2020	WIS	Native/Exotic	Conserv. Status	Growth Habit	Coef. of Conserv.
Aceraceae	*Acer negundo* L. var. *negundo*		X	4%	i	i	FAC	N		Tree	1
Aceraceae	*Acer saccharinum* L.		X	i	i	i	FAC	N		Tree	4
Alismataceae	*Alisma triviale* ​Pursh		X	1989			OBL	N		Forb	4
Alismataceae	^∼^*Sagittaria brevirostra* Mack. & Bush		X			10%	OBL	N		Forb	4
Alismataceae	*Sagittaria calycina* Engelm. var. *calycina*			i			OBL	N		Forb	3
Alismataceae	*Sagittaria cuneata* Sheldon				i		OBL	N		Forb	5
Amaranthaceae	*Amaranthus arenicola* I.M. Johnst.		X				FACU	N		Forb	1
Amaranthaceae	*Amaranthus retroflexus* L.		X	i	8%	20%	FACU	N		Forb	0
Amaranthaceae	*Amaranthus tuberculatus* ​(Moq.) Sauer					i	FAC	N		Forb	0
Amaranthaceae	*Froelichia gracilis* (Hook.) Moq.				i			N		Forb	3
Anacardiaceae	*Rhus glabra* L.		X		4%		UPL^1^	N		Shrub	2
Anacardiaceae	*Rhus trilobata* Nutt.	Hall			4%	10%		N		Shrub	4
Anacardiaceae	*Toxicodendron radicans* (L.) Kuntze var. *radicans*		X	22%	35%	i	FACU	N		Shrub	2
Apiaceae	*Cicuta maculata* L.		X	4%	i	i	OBL	N		Forb	5
Apiaceae	*Conium maculatum* L.			4%	i	30%	FACW	E		Forb	
Apiaceae	*Daucus carota* L.		X	i		i	UPL	E		Forb	
Apiaceae	*Sanicula canadensis* L. var. *canadensis*		X	i	4%	i	FACU	N		Forb	3
Apiaceae	*Sium suave* Walter		X	4%	i		OBL	N		Forb	7
Apocynaceae	*Apocynum cannabinum* L.		X	65%	42%	30%	FAC	N		Forb	2
Asclepiadaceae	*Asclepias arenaria* Torr.				i			N		Forb	5
Asclepiadaceae	*Asclepias incarnata* L. ssp. *incarnata*		X	26%	15%	30%	FACW	N		Forb	4
Asclepiadaceae	*Asclepias speciosa* Torr.		X	9%	27%	20%	FAC	N		Forb	1
Asclepiadaceae	*Asclepias stenophylla* A. Gray				i			N		Forb	6
Asclepiadaceae	*Asclepias sullivantii* Engelm. e*x* A. Gray			4%		20%	FAC	N		Forb	7
Asclepiadaceae	*Asclepias syriaca* L.		X	39%	15%	40%	UPL	N		Forb	1
Asclepiadaceae	*Asclepias verticillata* L.		X	22%	50%	70%	FACU	N		Forb	3
Asclepiadaceae	*Asclepias viridiflora* Raf.				i			N		Forb	6
Asteraceae	*Achillea millefolium* L.		X	4%	8%	40%	FACU	B		Forb	2
Asteraceae	*Ambrosia artemisiifolia* L.		X	52%	31%	60%	FACU	B		Forb	0
Asteraceae	*Ambrosia psilostachya* D.C.		X	100%	88%	90%	FACU	N		Forb	1
Asteraceae	*Ambrosia trifida* L. var. *trifida*		X	13%	i	40%	FAC	N		Forb	0
Asteraceae	*Antennaria neglecta* Greene		X	9%	4%	20%	FACU	N		Forb	3
Asteraceae	*Arctium minus* Bernh.		X	4%	4%	10%	FACU	E		Forb	
Asteraceae	*Artemisia ludoviciana* Nutt. Var. *ludoviciana*		X	i	27%	30%	UPL	B		Forb	4
Asteraceae	*Symphyotrichum ericoides* (L.) G.L. Nesom ssp. e*ricoides*		X	83%	81%	50%	FACU	N		Forb	3
Asteraceae	*Symphyotrichum lanceolatum* (Willd.) G.L. Nesom ssp. *lanceolatum*		X	48%	23%	10%	FACW	N		Forb	2
Asteraceae	*Symphyotrichum novaeangliae* (L.) G.L. Nesom			4%	4%	10%	FACW	N		Forb	4
Asteraceae	*Symphyotrichum praealtum* (Poir.) G.L. Neson var. *nebraskense* (Britton) G.L. Nesom		X	65%	42%	50%	FACW	N		Forb	5
Asteraceae	*Bidens bipinnata* L.			4%		10%	FACU	N		Forb	0
Asteraceae	*Bidens cernua* L.		X		4%		OBL	N		Forb	3
Asteraceae	*Bidens tripartita* L.		X	i		10%	FACW	N		Forb	2
Asteraceae	*Bidens frondosa* L.		X	i			FACW	N		Forb	4
Asteraceae	*Brickellia eupatorioides* (L.) Shinners var. *crymbulosa* (Torr. & A. Gray) Shinners				i	10%		N		Forb	4
Asteraceae	*Carduus nutans* L.		X	i	4%	20%	FACU	E^2^		Forb	
Asteraceae	*Cirsium altissimum* (L.) Hill			43%	35%	40%	FACU^1^	N		Forb	1
Asteraceae	*Cirsium arvense* (L.) Scop.	Hall		17%	4%	20%	FACU	E^2^		Forb	
Asteraceae	*Cirsium flodmanii* (Rydb.) Arthur			35%	19%	30%	FAC	N		Forb	4
Asteraceae	*Cirsium undulatum* (Nutt.) Spreng.			4%	4%		FACU	N		Forb	4
Asteraceae	*Cirsium vulgare* (Savi) Ten.			17%	12%	20%	UPL	E		Forb	
Asteraceae	*Conyza canadensis* (L.) Cronquist			39%	50%	60%	FAC^1^	N		Forb	0
Asteraceae	*Coreopsis tinctoria* Nutt. var. *tinctoria*		X	i		20%	FAC	N		Forb	1
Asteraceae	*Crepis runcinata* (James) Torr. & A. Gray var. *runcinata*		X	4%			FAC	N		Forb	5
Asteraceae	*Echinacea angustifolia* DC. var. *angustifolia*				i	20%		N		Forb	5
Asteraceae	*Eclipta prostrata* (L.) L.			i		10%	FACW	N		Forb	2
Asteraceae	*Erechtites hieraciifolius* (L.) Raf. e*x* DC.	Hall		4%	4%		FACW^1^	N		Forb	1
Asteraceae	*Erigeron philadelphicus* L.		X	i	8%	i	FAC	N		Forb	3
Asteraceae	*Erigeron strigosus* Muhl. e*x* Willd.		X	22%	19%	40%	FACU	N		Forb	2
Asteraceae	*Eupatorium altissimum* L.			9%	12%	40%	FACU^1^	N		Forb	3
Asteraceae	*Eupatorium perfoliatum* L.		X	13%	4%		FACW	N		Forb	5
Asteraceae	*Euthamia gymnospermoides* Greene		X	26%	4%		FAC	N		Forb	4
Asteraceae	*Pseudognaphalium obtusifolium* (L.) Hilliard & B.L. Burtt			4%	i			N		Forb	3
Asteraceae	*Grindelia squarrosa* (Pursh) Dunal		X	17%	8%	i	UPL	N		Forb	1
Asteraceae	*Helianthus annuus* L.		X	4%	12%	30%	FACU	N		Forb	0
Asteraceae	*Helenium autumnale* L.		X	35%	12%	10%	FACW	N		Forb	6
Asteraceae	*Helianthus grosseserratus* M. Martens		X	17%	4%	20%	FACW	N		Forb	4
Asteraceae	*Heliopsis helianthoides* (L.) Sweet		X	i		30%	FACU	N		Forb	4
Asteraceae	*Helianthus maximiliani* Schrad.		X	74%	27%	40%	FACU	N		Forb	4
Asteraceae	*Helianthus pauciflorus* Nutt.				8%	40%	FACU^1^	N		Forb	5
Asteraceae	*Helianthus petiolaris* Nutt.		X	13%	4%	20%		N		Forb	1
Asteraceae	*Helianthus tuberosus* L.		X	i	i	i	FACU	N		Forb	4
Asteraceae	*Heterotheca subaxillaris* (Lam.) Britton & Rusby	Hall			i	10%		N		Forb	2
Asteraceae	*Heterotheca villosa* (Pursh) Shinners		X	4%	12%			N		Forb	4
Asteraceae	*Iva annua* L.		X	13%	31%	20%	FAC	N		Forb	1
Asteraceae	*Cyclachaena xanthiifolia* (Nutt.) Fresen		X		i	i	FAC	N		Forb	0
Asteraceae	*Lactuca canadensis* L.		X				FACU	N		Forb	2
Asteraceae	*Lactuca serriola* L.		X	17%	4%	30%	FAC	E		Forb	
Asteraceae	*Lactuca tatarica* (L.) C.A. Mey. var. *pulchella* (Pursh) Breitung		X				UPL	N		Forb	
Asteraceae	*Leucanthemum vulgare* Lam.		X	9%			UPL	E		Forb	
Asteraceae	*Liatris glabarata* Rydb.				i			N		Forb	5
Asteraceae	*Liatris lancifolia* (Greene) Kittell			17%	4%	40%	FAC	N		Forb	8
Asteraceae	*Liatris punctata* Hook.		X					N		Forb	5
Asteraceae	*Liatris pycnostachya* Michx.		X				FAC	N		Forb	7
Asteraceae	*Lygodesmia juncea* (Pursh) D. Don *ex* Hook.				4%			N		Forb	4
Asteraceae	*Matricaria discoidea* DC.			i	i	i	FACU	E		Forb	
Asteraceae	*Packera plattensis* (Nutt.) W.A. Weber & Á. Löve		X	13%	8%	10%	FACU	N		Forb	5
Asteraceae	*Ratibida columnifera* (Nutt.) Wooton & Standl.		X	17%	46%	50%	FACU^1^	N		Forb	4
Asteraceae	*Ratibida pinnata* (Vent.) Barnhart	Hall		i	i	i		N		Forb	4
Asteraceae	*Rudbeckia hirta* L.		X	30%	27%	60%	FACU	N		Forb	4
Asteraceae	*Rudbeckia laciniata* L.			4%	4%		FAC	N		Forb	4
Asteraceae	*Silphium integrifolium* Michx. var. *laeve* Torr. & A. Gray	Hall	X		4%	30%	FAC	N		Forb	4
Asteraceae	*Silphium laciniatum* L.				i			N		Forb	5
Asteraceae	*Solidago canadensis* L.		X	87%	69%	50%	FACU	N		Forb	2
Asteraceae	*Solidago gigantea* Aiton		X	26%	12%	20%	FAC	N		Forb	3
Asteraceae	*Solidago missouriensis* Nutt.		X	13%	23%	10%		N		Forb	5
Asteraceae	*Oligoneuron rigidum* (L.) Small		X	22%	27%	30%	FACU	N		Forb	3
Asteraceae	*Sonchus asper* (L.) Hill	Hall	X			10%	FAC	E		Forb	
Asteraceae	*Taraxacum officinale* F.H. Wigg.		X	43%	31%	70%	FACU	E		Forb	
Asteraceae	*Tragopogon dubius* Scop.		X	i	4%	20%		E		Forb	
Asteraceae	*Vernonia baldwinii* Torr.		X	4%	4%	10%	FACU	N		Forb	3
Asteraceae	*Vernonia fasciculata* Michx.		X	48%	54%	30%	FAC	N		Forb	4
Asteraceae	*Xanthium strumarium* L.		X	4%	4%	10%	FAC	N		Forb	1
Bignoniaceae	*Catalpa speciosa* (Warder) Warder *ex* Engelm.			4%			FACU	N		Tree	
Boraginaceae	*Cynoglossum officinale* L.	Hall			4%		FACU	E^2^		Forb	
Boraginaceae	*Hackelia virginiana* (L.) I.M. Johnst.	Hall		13%	8%		FACU	N		Forb	2
Boraginaceae	*Lithospermum incisum* Lehm.		X	4%	27%	30%	UPL^1^	N		Forb	5
Boraginaceae	*Onosmodium bejariense* DC. e*x* A. DC. var. *occidentale* (Mack.) B.L. Turner		X		35%		FACU^1^	N		Forb	4
Brassicaceae	*Alliaria petiolata* (M. Bieb.) Cavara & Grande				i		FACU	E^2^		Forb	
Brassicaceae	*Turritis glabra* L.	Hall	X		i			N		Forb	7
Brassicaceae	*Arabis hirsuta* (L.) Scop. var. *pycnocarpa* (M. Hopkins) Rollins				i		FACU	N		Forb	5
Brassicaceae	*Capsella bursapastoris* (L.) Medik.		X	i	4%	i	FACU	E		Forb	
Brassicaceae	*Chorispora tenella* (Pall.) DC.		X	i	i			E		Forb	
Brassicaceae	*Descurainia pinnata* (Walter) Britton		X	i	i			N		Forb	0
Brassicaceae	*Descurainia sophia* (L.) Webb ex Prantl		X					E		Forb	
Brassicaceae	*Draba reptans* (Lam.) Fernald			i				N		Forb	
Brassicaceae	*Hesperis matronalis* L.	Hall			i	i	FACU	E		Forb	
Brassicaceae	*Lepidium densiflorum* Schrad.		X	9%	8%	10%	FAC	B		Forb	0
Brassicaceae	*Rorippa curvipes* Greene var. *truncata* (Jeps.) Rollins	Hall		4%	i	i	OBL	N		Forb	3
Brassicaceae	*Rorippa palustris* (L.) Besser var. *glabra* (O. E. Schulz) Roy L. Taylor & MacBryde		X		i	10%	OBL	N		Forb	4
Brassicaceae	*Sisymbrium loeselii* L.		X	4%	i	i		E		Forb	
Brassicaceae	*Thlaspi arvense* L.		X	13%	8%	10%	FACU	E		Forb	
Cactaceae	*Opuntia fragilis* (Nutt.) Haw.	Hall			8%	10%	UPL^1^	N		Shrub	3
Cactaceae	*Opuntia humifusa* (Raf.) Raf.				i			N		Shrub	5
Campanulaceae	*Campanula aparinoides* Pursh			9%	8%		OBL	N		Forb	7
Campanulaceae	*Lobelia cardinalis* L.	Hall	X	i	4%	10%	FACW	N	T2	Forb	6
Campanulaceae	*Lobelia siphilitica* L.		X	22%	4%	10%	OBL	N		Forb	6
Campanulaceae	*Lobelia spicata* Lam.		X	4%	4%	30%	FAC	N		Forb	6
Campanulaceae	*Triodanis perfoliata* (L.) Nieuwl.		X	4%		i	FAC	N		Forb	2
Cannabaceae	*Cannabis sativa* L.		X	13%	15%	10%		E		Forb	
Capparaceae	*Cleome serrulata* Pursh		X		i		FACU	N		Forb	0
Caprifoliaceae	*Sambucus nigra* L. ssp. *canadensis* (L.) R. Bolli		X	i		i		B		Shrub	2
Caprifoliaceae	*Symphoricarpos occidentalis* Hook.		X	4%	42%	10%	UPL	N		Shrub	2
Caprifoliaceae	*Symphoricarpos orbiculatus* Moench			9%	15%	10%	FACU	N		Shrub	2
Caryophyllaceae	*Cerastium brachypodum* (Engelm. ex A. Gray) B.L. Rob.				4%		FACU	N		Forb	1
Caryophyllaceae	*Dianthus armeria* L.	Hall		i	i		UPL	E		Forb	
Caryophyllaceae	*Holosteum umbellatum* L.			i		i		E		Forb	
Caryophyllaceae	*Silene antirrhina* L.		X	i	4%	10%		N		Forb	2
Caryophyllaceae	*Stellaria media* (L.) Vill.	Hall		i		10%	FACU	E		Forb	
Celastraceae	*Celastrus scandens* L.		X		i		UPL	N		Vine	4
Chenopodiaceae	*Chenopodium album* L.		X	9%	i	20%	FACU	B		Forb	
Chenopodiaceae	*Chenopodium berlandieri* Moq. var. *zschackii* (Murr) Murr ex Asch.			4%	8%			N		Forb	0
Chenopodiaceae	*Chenopodium glaucum* L. var. *glaucum*		X	i		i	FAC	E		Forb	
Chenopodiaceae	*Chenopodium missouriense* Aellan		X	4%		10%		N		Forb	0
Chenopodiaceae	*Chenopodium pratericola* Rydb.			9%	4%			N		Forb	1
Chenopodiaceae	*Chenopodium simplex* (Torr.) Raf.			4%	4%			N		Forb	1
Chenopodiaceae	*Chenopodium standleyanum* Aellan		X	4%	4%			N		Forb	4
Chenopodiaceae	*Cycloloma atriplicifolium* (Spreng.) J.M. Coult.		X		i		FACU	N		Forb	2
Chenopodiaceae	*Kochia scoparia* (L.) A.J. Scott		X	9%	i	i	FACU	E		Forb	
Clusiaceae	*Hypericum perforatum* L.					10%	UPL	E^2^		Forb	
Commelinaceae	*Commelina communis* L.			i			FAC	E		Forb	
Commelinaceae	*Tradescantia bracteata* Small		X	4%	i		FACU	N		Forb	5
Commelinaceae	*Tradescantia occidentalis* (Britton) Smyth		X	i	i		UPL	N		Forb	5
Convolvulaceae	*Calystegia macounii* (Greene) Brummitt			4%				N		Vine	5
Convolvulaceae	*Calystegia sepium* (L.) R. Br.		X	4%	8%	i	FAC	B		Vine	1
Convolvulaceae	*Convolvulus arvensis* L.		X	4%	8%	30%		E		Vine	
Convolvulaceae	*Ipomoea purpurea* (L.) Roth		X	i			FACU	E		Vine	
Cornaceae	*Cornus drummondii* C.A. Mey		X	22%	46%	40%	FAC	N		Shrub	3
Cornaceae	*Cornus sericea* L. ssp. *sericea*		X				FACW	N		Shrub	6
Crassulaceae	*Penthorum sedoides L.*			i			OBL	N		Forb	4
Cucurbitaceae	*Echinocystis lobata* (Michx.) Torr. & A. Gray		X		i		FAC	N		Vine	3
Cupressaceae	*Juniperus virginiana L.*		X	4%	27%	i	UPL	N		Tree	1
Cuscutaceae	*Cuscuta glomerata* Choisy		X	43%	4%	10%		N		Vine	5
Cuscutaceae	*Cuscuta gronovii* Willd. ex Schult.	Hall		4%				N	T2	Vine	6
Cyperaceae	*Bolboschoenus fluviatilis* (Torr.) Soják		X	i	4%	10%	OBL	N		Gram.	3
Cyperaceae	*Bolboschoenus maritimus* (L.) Palla ssp. *palludosus* (A. Nelson) T. Koyama		X				OBL	N		Gram.	5
Cyperaceae	*Carex aurea* Nutt.	Hall				i	OBL	N		Gram.	7
Cyperaceae	*Carex bicknellii* Britton	Hall			8%		FACW	N		Gram.	6
Cyperaceae	*Carex blanda* Dewey	Hall	X		4%		FAC	N		Gram.	2
Cyperaceae	*Carex brevior* (Dewey) Mack.		X	48%	69%	50%	FAC	N		Gram.	4
Cyperaceae	*Carex crawei* Dewey				i	i	FACW	N		Gram.	6
Cyperaceae	*Carex duriuscula* C.A. Mey.		X	4%	4%			N		Gram.	2
Cyperaceae	^+^*Carex emoryi* Dewey			9%	8%	10%	OBL	N		Gram.	5
Cyperaceae	*Carex gravida* L.H. Bailey		X	17%	8%	i	FACW	N		Gram.	4
Cyperaceae	*Carex granularis* Muhl. e*x* Willd.	Hall		4%			OBL	N		Gram.	6
Cyperaceae	*Carex hallii* Olney		X	i	i		FAC	N		Gram.	7
Cyperaceae	*Carex inops* L.H. Bailey ssp. *heliophila* (Mack.) Crins				2000			N		Gram.	5
Cyperaceae	*Carex interior* L.H. Bailey			2000			OBL	N		Gram.	7
Cyperaceae	*Carex laeviconica* C. Dewey				i		OBL	N		Gram.	4
Cyperaceae	*Carex meadii* Dewey		X	4%	4%	10%	FAC	N		Gram.	6
Cyperaceae	*Carex molesta* Mack. ex *Bright*		X	i	i		FACW	N		Gram.	3
Cyperaceae	*Carex pellita* Muhl. e*x* Willd.		X	30%	31%	10%	OBL	N		Gram.	4
Cyperaceae	*Carex praegracilis* W. Boott		X	43%	35%	40%	FACW	N		Gram.	4
Cyperaceae	*Carex sartwellii* Dewey			i	i		FACW	N		Gram.	6
Cyperaceae	*Carex scoparia* Schkuhr *ex* Willd.		X	4%			FACW	N		Gram.	5
Cyperaceae	*Carex stipata* Muhl. e*x* Willd.		X	i			OBL	N		Gram.	5
Cyperaceae	*Carex tetanica* Schkuhr			9%	4%		FACW	N		Gram.	7
Cyperaceae	*Carex vulpinoidea* Michx.		X	13%	12%	i	FACW	N		Gram.	4
Cyperaceae	*Carex spp.*		X	96%	100%	70%		N		Gram.	
Cyperaceae	*Cyperus acuminatus* Torr. & Hook. ex Torr.			i			OBL	N		Gram.	3
Cyperaceae	*Cyperus bipartitus* Torr.	Hall			4%	i	FACW	N		Gram.	5
Cyperaceae	*Cyperus diandrus* Torr.			i		10%	FACW	N		Gram.	5
Cyperaceae	*Cyperus erythrorhizos* Muhl.	Buffalo/Hall			4%	10%	OBL	N		Gram.	4
Cyperaceae	*Cyperus esculentus* L. var. *leptostachyus* Boeck.		X	4%	15%	20%	FACW	B		Gram.	0
Cyperaceae	*Cyperus fuscus* L.			4%			FACW	E		Gram.	
Cyperaceae	*Cyperus lupulinus* (Spreng.) Marcks ssp. *Lupulinus*			22%	31%	20%	FACU	N		Gram.	1
Cyperaceae	*Cyperus odoratus* L. var. *squarrosus* (Britt.) S. Jones, Wippff, & R. Carter		X	13%	4%	10%	FACW	N		Gram.	3
Cyperaceae	*Cyperus schweinitzii* Torr.		X	13%	31%		FACU	N		Gram.	4
Cyperaceae	*Cyperus squarrosus* L.		X	i	i		OBL	N		Gram.	2
Cyperaceae	*Cyperus strigosus* L.			4%		10%	FACW	N		Gram.	4
Cyperaceae	*Eleocharis acicularis* (L.) Roem. & Schult.		X	i			OBL	N		Gram.	4
Cyperaceae	*Eleocharis compressa* Sull.		X	9%	12%	10%	FACW	N		Gram.	6
Cyperaceae	*Eleocharis engelmannii* Steud.			i			FACW	N		Gram.	3
Cyperaceae	*Eleocharis erythropoda* Steud.			65%	62%	50%	FACW^1^	N		Gram.	5
Cyperaceae	*Eleocharis macrostachya* Britton		X	48%	46%	40%	OBL	N		Gram.	4
Cyperaceae	*Eleocharis palustris* (L.) Roem. & Schult.			30%	35%	30%	OBL	N		Gram.	4
Cyperaceae	*Fimbristylis puberula* (Michx.) Vahl var. *interior* (Britton) Kral		X	17%	12%		OBL	N		Gram.	7
Cyperaceae	*Fuirena simplex* Vahl var. *aristulata* (Torr.) Kral			2000	4%	10%	OBL	N		Gram.	6
Cyperaceae	*Lipocarpha aristulata* (Coville) G. Tucker			i	8%	10%	FACW	N		Gram.	6
Cyperaceae	*Schoenoplectus acutus* (Muhl. ex Bigelow) Á. Löve & D. Löve			i	4%	10%	OBL	N		Gram.	5
Cyperaceae	*Schoenoplectus pungens* (Vahl) Palla		X	65%	58%	30%	OBL	N		Gram.	4
Cyperaceae	*Schoenoplectus tabernaemontani* (C.C. Gmel.) Palla		X	i	i	i	OBL	N		Gram.	5
Cyperaceae	*Scirpus atrovirens* Willd.	Hall	X	i		10%	OBL	N		Gram.	5
Cyperaceae	*Scirpus pallidus* (Britton) Fernald			9%		10%	OBL	N		Gram.	5
Dryopteridaceae	*Onoclea sensibilis* L.	Hall		4%			FACW	N		Forb	7
Elaeagnaceae	*Elaeagnus angustifolia* L.		X	i	i	i	FACU	E		Shrub	
Elaeagnaceae	*Shepherdia argentea* (Pursh) Nutt.		X		i		UPL	N		Shrub	4
Equisetaceae	*Equisetum arvense* L.		X	13%	8%	10%	FAC	N		Forb	4
Equisetaceae	*Equisetum hyemale* L.					10%	FACW	N		Forb	4
Equisetaceae	*Equisetum laevigatum* A. Braun		X	78%	77%	30%	FAC	N		Forb	4
Equisetaceae	*Equisetum ×ferrissii* Clute (pro sp.) *[hyemale × laevigatum]*			i	8%		FACW	N		Forb	4
Euphorbiaceae	*Croton capitatus* Michx. var. *capitatus*	Hall			i			N		Forb	1
Euphorbiaceae	*Croton glandulosus* L. var. *septentrionalis* Müll. Arg.	Hall			i			B		Forb	1
Euphorbiaceae	*Croton texensis* (Klotzsch) Müll. Arg. var. *texensis*		X	4%	31%	10%	UPL^1^	N		Forb	1
Euphorbiaceae	*ˆEuphorbia davidii* Subils			4%	8%			E		Forb	0
Euphorbiaceae	*Euphorbia esula* L.				i			E^2^		Forb	
Euphorbiaceae	*Chamaesyce glyptosperma* (Engelm.) Small		X	13%	8%			N		Forb	0
Euphorbiaceae	*Chamaesyce maculata* (L.) Small				i	i	FACU	N		Forb	0
Euphorbiaceae	*Euphorbia marginata* Pursh		X		8%	10%	FACU	E		Forb	0
Euphorbiaceae	*Chamaesyce serpyllifolia* (Pers.) Small	Hall		4%	8%			N		Forb	2
Euphorbiaceae	*Euphorbia spathulata* Lam.				i		FACU	N		Forb	2
Euphorbiaceae	*Chamaesyce stictospora* (Engelm.) Small			4%	8%	10%		N		Forb	0
Fabaceae	*Amorpha canescens* Pursh					10%		N		Shrub	6
Fabaceae	*Amorpha fruticosa* L.		X	22%	4%	i	FACW	N		Shrub	5
Fabaceae	*Amphicarpaea bracteata* (L.) Fernarld var. *comosa*				4%			N		Vine	4
Fabaceae	*Apios americana* Medik.		X				FAC	N		Forb	6
Fabaceae	*Astragalus canadensis* L.		X			40%	FAC	N		Forb	5
Fabaceae	*Astragalus plattensis* Nutt.	Hall				10%		N		Forb	7
Fabaceae	*Chamaecrista fasciculata* (Michx.) Greene					10%	FACU	N		Forb	1
Fabaceae	*Dalea candida* Michx. ex Willd.		X	30%	23%	40%		N		Forb	6
Fabaceae	*Dalea leporina* (Aiton) Bullock					i	UPL	N		Forb	3
Fabaceae	*Dalea purpurea* Vent.		X	30%	23%	30%	FAC^1^	N		Forb	6
Fabaceae	*Desmodium canadense* (L.) DC.					10%	FAC	N		Forb	5
Fabaceae	*Desmodium glutinosum* (Muhl. ex Willd.) Alph. Wood		X		4%			N		Forb	
Fabaceae	*Desmodium illinoense* A. Gray				4%	30%		N		Forb	6
Fabaceae	*Desmanthus illinoensis* (Michx.) MacMill. ex B.L. Rob. & Fernald		X	39%	38%	70%	FACU	N		Forb	5
Fabaceae	*Gleditsia triacanthos* L.		X	4%	15%	i	FACU	N		Tree	1
Fabaceae	*Glycyrrhiza lepidota* Pursh		X	43%	35%	40%	FACU	N		Forb	4
Fabaceae	*Gymnocladus dioicus* (L.) K. Koch				i			N		Tree	5
Fabaceae	*Lespedeza capitata* Michx.		X	i	4%	10%	UPL	N		Forb	5
Fabaceae	*Lotus corniculatus* L.	Hall	X	4%			FACU	E		Forb	
Fabaceae	*Lotus unifoliolatus* (Hook.) Benth.		X	17%	8%	10%		N		Forb	3
Fabaceae	*Lotus tenuis* Waldst. & Kit. ex Willd.			13%		i	FACU	E		Forb	
Fabaceae	*Medicago lupulina* L.		X	65%	73%	90%	FACU	E		Forb	
Fabaceae	*Medicago sativa* L.		X	i	i	10%	UPL	E		Forb	
Fabaceae	*Melilotus albus* Medik.		X	52%	54%	90%	FACU	E		Forb	
Fabaceae	*Melilotus officinalis* (L.) Lam.		X	22%	27%	50%	FACU	E		Forb	
Fabaceae	*Mimosa nuttallii* (DC. ex Britton & Rose) B.L. Turner			4%	i	40%		N		Forb	6
Fabaceae	*Oxytropis lambertii* Pursh					10%	UPL	N		Forb	6
Fabaceae	*Pediomelum argophyllum* (Pursh) J. Grimes			4%		10%		N		Forb	6
Fabaceae	*Robinia pseudoacacia* L.	Hall		i	4%	i	UPL	N		Tree	
Fabaceae	*Securigera varia* (L.) Lassen	Hall		i				E^2^		Forb	
Fabaceae	*Strophostyles leiosperma* (Torr. & A. Gray) Piper		X	4%	31%	10%		N		Forb	4
Fabaceae	*Trifolium hybridum* L.		X	i			FACU	E		Forb	
Fabaceae	*Trifolium fragiferum* L.			i		i	FAC	E		Forb	
Fabaceae	*Trifolium pratense* L.		X	39%	35%	20%	FACU	E		Forb	
Fabaceae	*Trifolium repens* L.		X	17%	8%	10%	FACU	E		Forb	
Fabaceae	*Vicia villosa* Roth	Hall			i	10%		E		Forb	
Fumariaceae	*Corydalis micrantha* (Engelm. ex A. Gray) A. Gray ssp. *micrantha*	Hall		i				N		Forb	0
Fumariaceae	*Fumaria officinalis* L. ssp. *wirtgenii* (W. D. J. Koch) Arcang.	Hall			i			E		Forb	
Gentianaceae	*Centaurium pulchellum* (SW.) Druce	Hall		i			FACU	E		Forb	
Gentianaceae	*Eustoma exaltatum* (L.) Salisb. ex G. Don ssp. *russellianum* (Hook.) Kartesz		X	13%	23%	20%		N		Forb	4
Grossulariaceae	*Ribes missouriense* Nutt.				4%			N		Shrub	4
Hydrophyllaceae	*Ellisia nyctelea* (L.) L.		X		i		FAC	N		Forb	0
Iridaceae	*Belamcanda chinensis* (L.) DC.	Hall			i			E		Forb	
Iridaceae	*Iris germanica* L.	Hall			i			E		Forb	
Iridaceae	*Iris pseudacorus* L.	Buffalo/Hall			i	i	OBL	E^2^		Forb	
Iridaceae	*Sisyrinchium campestre* E.P. Bicknell		X	9%	8%	20%	FACW^1^	E		Forb	5
Iridaceae	*Sisyrinchium montanum* Greene var. *montanum*		X			i	FAC	N		Forb	5
Juncaceae	*Juncus arcticus* Willd. ssp. *littoralis* (Engelm.) Hultén		X	17%	12%	20%	FACW	N		Gram.	6
Juncaceae	*Juncus bufonius* L.		X				OBL	N		Gram.	4
Juncaceae	*Juncus dudleyi* Wiegand		X	22%	12%	20%	FACW	N		Gram.	5
Juncaceae	*Juncus interior* Wiegand			26%	27%	20%	FACW	N		Gram.	4
Juncaceae	*Juncus nodosus* L.		X	17%	19%	20%	OBL	N		Gram.	6
Juncaceae	*Juncus tenuis* Willd.	Hall		30%	23%	20%	FAC	N		Gram.	3
Juncaceae	*Juncus torreyi* Coville		X	30%	31%	30%	FACW	N		Gram.	4
Juncaginaceae	*Triglochin maritima* L.		X	i			OBL	N		Gram.	5
Lamiaceae	*Glechoma hederacea* L.	Hall				i	FACU	E		Forb	
Lamiaceae	*Hedeoma hispida* Pursh		X	i	8%			N		Forb	2
Lamiaceae	*Lamium amplexicaule* L.			i	i	i		E		Forb	
Lamiaceae	*Leonurus cardiaca* L. ssp. c*ardiaca*			i	4%	i		E		Forb	
Lamiaceae	*Lycopus americanus* Muhl. ex W.P.C. Barton		X	39%	38%	10%	OBL	N		Forb	4
Lamiaceae	*Lycopus asper* Greene		X	22%	12%		OBL	N		Forb	5
Lamiaceae	*Lycopus uniflorus* Michx. var. *uniflorus*			4%			OBL	N		Forb	6
Lamiaceae	*Mentha arvensis* L.		X	22%	4%	10%	FACW	N		Forb	4
Lamiaceae	*Monarda citriodora* Cerv. *ex* Lag. var. *citriodora*	Hall				i		N		Forb	
Lamiaceae	*Monarda fistulosa* L. var. *mollis*		X		19%	40%	UPL	N		Forb	4
Lamiaceae	*Nepeta cataria* L.		X	4%	15%	10%	FACU	E		Forb	
Lamiaceae	*Physostegia virginiana* (L.) Benth. ssp. *virginiana*			9%		20%	FACW	N		Forb	7
Lamiaceae	*Prunella vulgaris* L.			9%	15%	i	FAC	N		Forb	4
Lamiaceae	*Pycnanthemum virginianum* ​(L.) T. Dur. & B.D. Jacks. ex B.L. Rob. & Fernald		X	9%	12%		FAC	N		Forb	6
Lamiaceae	*Salvia azurea* Michx. ex Lam. var. *grandiflora* Benth.	Hall			i			N		Forb	6
Lamiaceae	*Scutellaria galericulata* L.	Hall		i			OBL	N		Forb	6
Lamiaceae	*Scutellaria lateriflora* L. var. *lateriflora*		X	4%	4%		FACW	N		Forb	6
Lamiaceae	*Stachys pilosa* Nutt.	Hall	X				FACW	N		Forb	5
Lamiaceae	*Teucrium canadense* L. var. canadense		X	22%	23%	10%	FACW	N		Forb	4
Lilliaceae	*Allium canadense* L. var. *canadense*		X	17%	4%	10%	FACU	E		Forb	3
Lilliaceae	*Allium canadense* L. var. *lavendulare* (Bates) Ownbey & Aase		X	9%	4%	10%	FACW	N		Forb	7
Lilliaceae	*Asparagus officinalis L.*		X	i	i	i		N		Forb	
Lilliaceae	*Maianthemum stellatum* (L.) Link			13%	8%	10%		N		Forb	4
Lilliaceae	*Hypoxis hirsuta* (L.) Coville		X	i	4%	i		N		Forb	7
Linaceae	*Linum sulcatum* Riddell			i	4%	10%		N		Forb	6
Lythraceae	*Ammannia coccinea* Rottb.	Hall	X				OBL	N		Forb	4
Lythraceae	*Ammannia robusta* Heer & Regel			i	4%	10%	OBL	N		Forb	4
Lythraceae	*Lythrum alatum* Pursh var. *alatum*		X	17%	4%	20%	OBL	N		Forb	6
Lythraceae	*Lythrum salicaria* L.		X	39%	27%	20%	OBL	E^2^		Forb	
Lythraceae	*Rotala ramosior* (L.) Koehne	Hall		4%			OBL	N		Forb	7
Malvaceae	*Abutilon theophrasti* Medik.			i	4%	20%	UPL	E		Forb	
Malvaceae	*Callirhoe alcaeoides* (Michx.) A. Gray		X	i	8%	20%		N		Forb	5
Malvaceae	*Callirhoe involucrata* (Torr. & A. Gray) A. Gray var. *involucrata*		X	35%	58%	30%	FACU^1^	N		Forb	2
Malvaceae	*Hibiscus laevis* All.	Buffalo		i	i	i	OBL	N		Forb	4
Malvaceae	*Hibiscus trionum* L.		X	9%				E		Forb	
Malvaceae	*Malva neglecta* Wallr.		X	i				E		Forb	
Malvaceae	*Malva* x*henningii* Goldb. *(pusilla x neglecta)*	Hall			i			E		Forb	
Molluginaceae	*Mollugo verticillata* L.				4%		FAC	N		Forb	
Moraceae	*Morus alba* L.		X	13%	23%	10%	FACU	E		Tree	
Nyctaginaceae	*Mirabilis hirsuta* (Pursh) MacMill.		X	13%	8%	20%		N		Forb	5
Nyctaginaceae	*Mirabilis linearis* (Pursh) Heimerl var. *linearis*		X		4%	10%		N		Forb	4
Nyctaginaceae	*Mirabilis nyctaginea* (Michx.) MacMill.		X	4%	i	i	UPL	N		Forb	1
Oleaceae	*Fraxinus pennsylvanica* Marshall		X	13%	12%	i	FAC	N		Tree	2
Onagraceae	*Circaea lutetiana* L. ssp*. canadensis* (L.) Asch. & Magnus				4%			N		Forb	5
Onagraceae	*Oenothera curtiflora* W.L. Wagner & Hoch		X	4%	19%	30%	UPL	N		Forb	1
Onagraceae	*Ludwigia palustris* (L.) Elliott		X		i		OBL	N		Forb	5
Onagraceae	*Oenothera biennis* L. var. *canescens* Torr. & A. Gray		X	26%	12%	20%	FACU	N		Forb	1
Onagraceae	*Oenothera laciniata* Hill		X	i	i		FACU	N		Forb	1
Onagraceae	*Oenothera latifolia* (Rydb.) Munz		X	i				N		Forb	4
Onagraceae	*Calylophus serrulatus* (Nutt.) P.H. Raven			4%	4%	10%		N		Forb	5
Onagraceae	*Oenothera speciosa* Nutt.			9%				N		Forb	
Orchidaceae	*Platanthera praeclara* Sheviak & Bowles		X					N	T1	Forb	9
Orchidaceae	*Spiranthes cernua* (L.) Rich.	Hall	X				FACW	N		Forb	6
Orchidaceae	*Spiranthes magnicamporum* Sheviak			4%	19%	i	FAC	N		Forb	7
Oxalidaceae	*Oxalis dillenii* Jacq.		X	39%	46%	40%	FACU	N		Forb	0
Phrymaceae	*Phryma leptostachya* L.			4%	4%		FACU	N		Forb	5
Plantaginaceae	*Plantago eriopoda* Torr.		X	4%			FAC	N		Forb	5
Plantaginaceae	*Plantago lanceolata* L.					i	FAC	E		Forb	
Plantaginaceae	*Plantago major* L.		X	4%	4%	i	FAC	E		Forb	
Plantaginaceae	*Plantago patagonica* Jacq. *var. patagonica*		X	13%	23%	40%	UPL^1^	N		Forb	1
Plantaginaceae	*Plantago patagonica* Jacq. *var. spinulosa*	Hall				i		N		Forb	1
Plantaginaceae	*Plantago rugelii* Decne.		X				FAC	N		Forb	0
Plantaginaceae	*Plantago virginica* L.			i	4%		FACU	N		Forb	2
Poaceae	*Agropyron cristatum* (L.) Gaertn.					i		E		Gram.	
Poaceae	*Agrostis stolonifera* L.		X	87%	85%	50%	FACW	E		Gram.	
Poaceae	*Alopecurus aequalis* Sobol.		X	i	i	i	OBL	N		Gram.	6
Poaceae	*Alopecurus arundinaceus* Poir.			i	23%	i	FACW	E		Gram.	
Poaceae	*Alopecurus pratensis* L.	Hall		i	8%		FACW	E		Gram.	
Poaceae	*Andropogon gerardii* Vitman		X	74%	77%	90%	FACU	N		Gram.	5
Poaceae	*Aristida oligantha* Michx.		X		4%			N		Gram.	2
Poaceae	*Avena fatua* L.		X			i		E		Gram.	
Poaceae	*Bouteloua curtipendula* (Michx.) Torr.			9%	4%	20%		N		Gram.	5
Poaceae	*Bouteloua dactyloides* (Nutt.) J.T. Columbus			i	i	i	FACU	N		Gram.	2
Poaceae	*Bouteloua gracilis* (Willd. ex Kunth) Lag. ex Griffiths		X	13%	19%	20%	FACU^1^	N		Gram.	4
Poaceae	*Bouteloua hirsuta* Lag.		X	4%				N		Gram.	6
Poaceae	*Bromus inermis* Leyss.		X	70%	65%	70%	UPL	B		Gram.	
Poaceae	*Bromus arvensis* L.		X	48%	38%	70%	FACU	E		Gram.	
Poaceae	*Bromus latiglumis* (Shear) Hitchc.			4%	4%		FACW	N		Gram.	5
Poaceae	*Bromus squarrosus* L.	Hall				10%		E		Gram.	
Poaceae	*Bromus tectorum* L.		X	13%	8%	20%		E		Gram.	
Poaceae	*Calamagrostis canadensis* (Michx.) P. Beauv.	Hall		i			OBL	N		Gram.	6
Poaceae	*Calamovilfa longifolia* (Hook.) Scribn.		X	13%	23%	10%	UPL^1^	N		Gram.	5
Poaceae	*Calamagrostis stricta* (Timm) Koeler		X	39%	35%	20%	FACW	N		Gram.	6
Poaceae	*Cenchrus longispinus* (Hack.) Fernald		X	13%	4%	i	UPL	N		Gram.	0
Poaceae	*Chloris verticillata* Nutt.		X	4%	27%	10%	UPL^1^	N		Gram.	0
Poaceae	*Dactylis glomerata* L.			i	19%	30%	FACU	E		Gram.	
Poaceae	*Digitaria cognata* (Schult.) Pilg.			26%	35%	20%	UPL^1^	N		Gram.	4
Poaceae	*Digitaria sanguinalis* (L.) Scop.		X	4%	8%	10%	FACU	E		Gram.	
Poaceae	*Distichlis spicata* (L.) Greene		X	26%	19%	30%	FACW	N		Gram.	3
Poaceae	*Echinochloa crusgalli* (L.) P. Beauv.		X	4%	i	10%	FAC	E		Gram.	
Poaceae	*Echinochloa muricata* (P. Beauv.) Fernald			i		10%	FACW	N		Gram.	0
Poaceae	*Eleusine indica* (L.) Gaertn.		X		12%		FACU	E		Gram.	
Poaceae	*Elymus canadensis* L.		X	13%	12%	40%	FACU	N		Gram.	5
Poaceae	*Thinopyrum intermedium* (Host) Barkworth & D.R. Dewey				4%			E		Gram.	
Poaceae	*Thinopyrum ponticum* (Podp.) Z.W. Liu & R.C. Wang		X			i		E		Gram.	
Poaceae	x*Elyhordeum macounii* (Vasey) Barkworth & D.R. Dewey			i			FAC	N		Gram.	4
Poaceae	*Elymus repens* (L.) Gould		X	70%	38%	i	FACU	E		Gram.	
Poaceae	*Pascopyrum smithii* (Rydb.) Á. Löve		X	17%	15%	20%	FACU	N		Gram.	3
Poaceae	*Elymus trachycaulus* (Link) Gould ex Shinners		X	4%	4%		FACU	N		Gram.	5
Poaceae	*Elymus villosus* Muhl. ex Willd.	Hall	X	13%	12%	i	FACU	N		Gram.	5
Poaceae	*Elymus virginicus* L.		X	13%	8%	10%	FACW	N		Gram.	4
Poaceae	*Eragrostis cilianensis* (All.) Vign. ex Janchen		X	i	4%	i	FACU	E		Gram.	
Poaceae	*Eragrostis pectinacea* (Michx.) Nees ex Steud.		X	i	4%	i	FAC	N		Gram.	0
Poaceae	*Eragrostis spectabilis* (Pursh) Steud.		X	39%	19%	10%	UPL	N		Gram.	3
Poaceae	*Eragrostis trichodes* (Nutt.) Alph. Wood			13%	15%	20%		N		Gram.	5
Poaceae	*Eriochloa villosa* (Thunb.) Kunth	Hall			i			E		Gram.	
Poaceae	*Festuca subverticillata* (Pers.) Alexeev	Hall			4%		FACU	N		Gram.	5
Poaceae	*Glyceria striata* (Lam.) Hitchc.	Hall	X	4%			OBL	N		Gram.	5
Poaceae	*Hesperostipa comata* (Trin. & Rupr.) Barkworth		X	4%	8%	10%	UPL^1^	N		Gram.	6
Poaceae	*Hesperostipa spartea* (Trin.) Barkworth		X					N		Gram.	6
Poaceae	*Hierochloe odorata* (L.) P. Beauv.		X					N		Gram.	
Poaceae	*Hordeum jubatum* L.		X	26%	8%	20%	FACW	N		Gram.	1
Poaceae	*Hordeum pusillum* Nutt.		X	i	i	i	FACU	N		Gram.	1
Poaceae	*Koeleria macrantha* (Ledeb.) Schult.		X	4%	4%	10%		N		Gram.	6
Poaceae	*Leersia oryzoides* (L.) Sw.			26%	12%	10%	OBL	N		Gram.	4
Poaceae	*Leersia virginica* Willd.		X	13%	4%	20%	FACW	N		Gram.	4
Poaceae	*Leptochloa fusca* (L.) Kunth		X	i		i	FACW	N		Gram.	1
Poaceae	*Muhlenbergia asperifolia* Nees & Meyen ex (Trin.) Parodi		X	70%	46%	10%	FACW	N		Gram.	5
Poaceae	*Muhlenbergia frondosa* (Poir.) Fernald				4%		FACW	N		Gram.	2
Poaceae	*Muhlenbergia mexicana* (L.) Trin.		X	13%	8%		FACW	N		Gram.	4
Poaceae	*Muhlenbergia racemosa* (Michx.) Britton, Sterns & Poggenb.		X	17%	4%		FACW	N		Gram.	4
Poaceae	*Muhlenbergia schreberi* J.F. Gmel.	Hall		4%	4%		FACU	N		Gram.	0
Poaceae	*∞Muhlenbergia sylvatica* (Torr.) Torr. ex A. Gray	Hall	X	4%	8%		FACW	N	T2	Gram.	6
Poaceae	^*>*^*Dichanthelium acuminatum* (Sw.) Gould & C.A. Clark		X	65%	46%	30%	FAC	N		Gram.	6
Poaceae	*Panicum capillare* L.		X	9%	23%	10%	FAC	N		Gram.	0
Poaceae	*Panicum dichotomiflorum* Michx.					i	FAC	N		Gram.	0
Poaceae	*Dichanthelium oligosanthes* (Schult.) Gould		X	57%	58%	50%	FACU^1^	N		Gram.	4
Poaceae	*Dichanthelium ovale* (Elliott) Gould & C.A. Clark				i		FACU	N		Gram.	6
Poaceae	*Panicum virgatum* L.		X	96%	88%	100%	FAC	N		Gram.	4
Poaceae	*Dichanthelium wilcoxianum* (Vasey) Freckmann			i				N		Gram.	7
Poaceae	*Paspalum setaceum* Michx.		X	26%	35%	20%	FACU	N		Gram.	2
Poaceae	*Phalaris arundinacea* L.		X	39%	50%	30%	FACW	N		Gram.	0
Poaceae	*Phleum pratense* L.		X	4%			FACU	E		Gram.	
Poaceae	*Phragmites australis* (Cav.) Trin. ex Steud.			9%	4%	20%	FACW	B^2^		Gram.	
Poaceae	*Poa arida* Vasey	Hall		i				N		Gram.	6
Poaceae	*Poa compressa* L.				8%		FACU	E		Gram.	
Poaceae	*Poa pratensis* L.		X	96%	92%	90%	FACU	B		Gram.	
Poaceae	*Polypogon monspeliensis* (L.) Desf.		X	i	i	i	FACW	E		Gram.	
Poaceae	*Puccinellia distans* (Jacq.) Parl.	Hall	X	1982			FACW	B		Gram.	
Poaceae	*Schedonorus arundinaceus* (Schreb.) Dumort.			22%	46%	40%	FACU	E		Gram.	
Poaceae	*Schedonorus pratensis* (Huds.) P. Beauv.		X	4%	19%	10%	FACU	E		Gram.	
Poaceae	*Schizachyrium scoparium* (Michx.) Nash		X	30%	46%	70%	FACU	N		Gram.	4
Poaceae	*Secale cereale* L.					i		E		Gram.	
Poaceae	*Setaria faberi* Herrm.					i	UPL	E		Gram.	
Poaceae	*Setaria pumila* (Poir.) Roem. & Schult.		X	22%	23%	30%	FACU	E		Gram.	
Poaceae	*Setaria verticillata* (L.) P. Beauv.	Hall			4%	10%	FAC	E		Gram.	
Poaceae	*Setaria viridis* (L.) P. Beauv.			4%	8%	40%		E		Gram.	
Poaceae	*Sorghastrum nutans* (L.) Nash		X	70%	65%	90%	FACU	N		Gram.	5
Poaceae	*Sorghum halepense* (L.) Pers.				i		FACU	E		Gram.	
Poaceae	*Spartina pectinata* Bosc ex Link		X	91%	85%	70%	FACW	N		Gram.	5
Poaceae	*Sphenopholis obtusata* (Michx.) Scribn.		X	4%			FAC	N		Gram.	5
Poaceae	*Sporobolus compositus* (Poir.) Merr.		X	65%	69%	70%	FACU^1^	N		Gram.	3
Poaceae	*Sporobolus cryptandrus* (Torr.) A. gray		X	35%	31%	30%	FACU	N		Gram.	2
Poaceae	*Sporobolus neglectus* Nash			17%	12%		UPL	N		Gram.	1
Poaceae	*Tripsacum dactyloides* (L.) L.	Hall		i			FAC	N		Gram.	7
Poaceae	*Tridens flavus* (L.) Hitchc.				8%		UPL	N		Gram.	2
Poaceae	*Triplasis purpurea* (Walter) Chapm.		X	4%	i			N		Gram.	5
Poaceae	*Vulpia octoflora* (Walter) Rydb.		X	i	4%		FACU	N		Gram.	3
Polygalaceae	*Polygala alba* Nutt.	Hall	X					N		Forb	5
Polygalaceae	*Polygala verticillata* L.					10%	FACU	N		Forb	4
Polygonaceae	*Eriogonum annuum* Nutt.				i			N		Forb	3
Polygonaceae	*Polygonum achoreum S.F. Blake*				i		FACU	N		Forb	0
Polygonaceae	*Polygonum arenastrum* Jord. ex Boreau		X	i	i	10%		E		Forb	0
Polygonaceae	*Polygonum amphibium* L. *var. emersum* Michx.		X	9%	8%	i	OBL	N		Forb	6
Polygonaceae	*Polygonum convolvulus* L.		X	9%	4%			E		Forb	
Polygonaceae	*Polygonum hydropiperoides* Michx.			i			OBL	N		Forb	6
Polygonaceae	*Polygonum lapathifolium* L.		X			i	OBL	N		Forb	2
Polygonaceae	*Polygonum pensylvanicum* L.			i		i	FACW	N		Forb	0
Polygonaceae	*Polygonum persicaria* L.		X	i	4%	i	FACW	E		Forb	
Polygonaceae	*Polygonum punctatum* Elliott		X	17%	8%	10%	OBL^1^	N		Forb	4
Polygonaceae	*Polygonum ramosissimum* Michx.			4%	4%		FACW	N		Forb	1
Polygonaceae	*Polygonum scandens* L.				4%	i		B		Forb	1
Polygonaceae	*Rumex acetosella* L.	Hall			i		FAC	E		Forb	
Polygonaceae	*Rumex altissimus* Alph. Wood				i	10%	FAC	N		Forb	0
Polygonaceae	*Rumex crispus* L.		X	9%	12%	20%	FAC	E		Forb	
Polygonaceae	*Rumex maritimus* L.			4%	i	10%	FACW	N		Forb	3
Polygonaceae	*Rumex stenophyllus* Ledeb.			4%	i	10%	FACW	E		Forb	
Pontederiaceae	*Heteranthera limosa* (Sw.) Willd.				i		OBL	N		Forb	4
Portulacaceae	*Phemeranthus parviflorus* (Nutt.) Kiger		X					N		Forb	7
Portulacaceae	*Portulaca oleracea* L.			i	i	i	FAC	B		Forb	0
Primulaceae	*Anagallis arvensis* L.	Hall		i			FACU	E		Forb	
Primulaceae	*Androsace occidentalis* Pursh				i		FACU	N		Forb	1
Primulaceae	*Lysimachia ciliata* L.		X	4%		i	FACW	N		Forb	5
Primulaceae	*Lysimachia thyrsiflora* L.		X	i	i		OBL	N		Forb	7
Ranunculaceae	*Anemone canadensis* L.		X	i	4%	i	FACW	N		Forb	4
Ranunculaceae	*Anemone caroliniana* Walter				i	10%		N		Forb	7
Ranunculaceae	*Anemone cylindrica* A. Gray				8%			N		Forb	4
Ranunculaceae	*Clematis virginiana* L.				12%		FAC	N		Vine	4
Ranunculaceae	*Delphinium carolinianum* Walter *ssp. virescens* (Nutt.) R.E. Brooks		X		i	i		N		Forb	6
Ranunculaceae	*Ranunculus abortivus* L.				4%		FAC	N		Forb	1
Ranunculaceae	*Ranunculus cymbalaria* Pursh		X	4%			OBL	N		Forb	3
Ranunculaceae	*Ranunculus longirostris* Godr.			i	i		OBL	N		Forb	6
Ranunculaceae	*Ranunculus macounii* Britton		X				OBL	N		Forb	5
Ranunculaceae	*Ranunculus sceleratus* L.			i		i	OBL	N		Forb	3
Ranunculaceae	*Thalictrum dasycarpum* Fisch. & AvéLall.		X				FAC	N		Forb	4
Rhamnaceae	*Rhamnus cathartica* L.	Hall				i	FACU	E		Tree	
Rosaceae	*Agrimonia parviflora* Aiton				12%		FACW	N		Forb	5
Rosaceae	*Fragaria virginiana* Duchesne					10%	FACU	N		Forb	5
Rosaceae	*Geum canadense* Jacq.		X	9%	15%	20%	FAC	N		Forb	3
Rosaceae	*Potentilla norvegica* L.		X	4%	i	10%	FAC	N		Forb	2
Rosaceae	*Potentilla paradoxa* Nutt.		X	4%	i	10%		N		Forb	4
Rosaceae	*Potentilla recta* L.				i	i		E^2^		Forb	
Rosaceae	*Prunus americana* Marshall		X	i	i	i	UPL	N		Shrub	3
Rosaceae	*Prunus virginiana* L.			i	i	i	FACU	N		Shrub	3
Rosaceae	*Rosa arkansana* Porter		X	17%	42%	20%	FACU	N		Shrub	4
Rosaceae	*Rosa woodsii* Lindl.		X		4%		FACU	N		Shrub	4
Rosaceae	*Rubus occidentalis* L.		X	i	19%			N		Shrub	3
Rubiaceae	*Galium aparine* L.		X		4%	i	FACU	N		Forb	0
Rubiaceae	*Galium circaezans* Michx. *var. hypomalacum* Fernald			i	8%		FACU	N		Forb	5
Rubiaceae	*Galium tinctorium* (L.) Scop.			4%			OBL	N		Forb	7
Rutaceae	*Zanthoxylum americanum* Mill.		X	4%	4%		UPL	N		Tree	4
Salicaceae	*Populus deltoides* W. Bartram ex Marshall		X	9%	12%	30%	FAC	N		Tree	3
Salicaceae	*Salix amygdaloides* Andersson		X	9%		i	FACW	N		Shrub	4
Salicaceae	*Salix eriocephala* Michx.					1985	FACW	N		Shrub	6
Salicaceae	*Salix exigua* ssp.*interior* (Rowlee) Cronquist		X	13%	23%	50%	FACW	N		Shrub	3
Scrophulariaceae	*Agalinis purpurea* (L.) Pennell			i			FACW	N	T2	Forb	8
Scrophulariaceae	*Agalinis tenuifolia* (Vahl) Raf.		X	17%	12%	10%	FAC	N		Forb	5
Scrophulariaceae	*Bacopa rotundifolia* (Michx.) Wettst.			1989			OBL	N		Forb	4
Scrophulariaceae	*Mimulus glabratus* Kunth var. *jamesii* (Torr. & A. Gray ex Benth.) A. Gray			4%			OBL	N		Forb	7
Scrophulariaceae	*Mimulus ringens* L.		X	i			OBL	N		Forb	6
Scrophulariaceae	*Penstemon albidus* Nutt.					10%		N		Forb	6
Scrophulariaceae	*Penstemon digitalis* Nutt. ex Sims	Hall				30%	FACW	N	T2	Forb	6
Scrophulariaceae	*Penstemon grandiflorus* Nutt.				4%	30%		N		Forb	5
Scrophulariaceae	*Penstemon tubaeflorus* Nutt. var. *tubaeflorus* Nutt.	Hall				30%		N	T2	Forb	6
Scrophulariaceae	*Veronica americana* Schwein. ex Benth.				4%		OBL	N		Forb	7
Scrophulariaceae	*Veronica anagallis-aquatica* L.	Hall	X	i		10%	OBL	N		Forb	
Scrophulariaceae	*Verbascum blattaria* L.	Buffalo				i	UPL	E		Forb	
Scrophulariaceae	*Veronica arvensis* L.	Hall			i			E		Forb	
Scrophulariaceae	*Veronica peregrina* L.			i			FACW	N		Forb	1
Scrophulariaceae	*Veronica polita* Fr.				i			E		Forb	
Scrophulariaceae	*Verbascum thapsus* L.		X	4%	4%	30%	UPL	E		Forb	
Smilacaceae	*Smilax hispida* L.		X	4%	4%		FAC	N		Vine	4
Solanaceae	*Datura stramonium* L.			i				E		Forb	
Solanaceae	*Physalis heterophylla* Nees		X	9%	27%	i	UPL^1^	N		Forb	4
Solanaceae	*Physalis hispida* (Waterf.) Cronquist			i	4%			N		Forb	4
Solanaceae	*Physalis longifolia* Nutt.			4%	8%	i		N		Forb	0
Solanaceae	*Physalis virginiana* Mill.		X	4%	23%	40%	FAC^1^	N		Forb	6
Solanaceae	*Solanum carolinense* L.			4%	8%	20%	UPL	N		Forb	2
Solanaceae	*Solanum interius* Rydb.		X					N		Forb	1
Solanaceae	*Solanum ptychanthum* Dunal		X	4%	15%	10%	FACU	N		Forb	0
Solanaceae	*Solanum rostratum* Dunal		X	13%	8%			N		Forb	0
Sparganiaceae	*Sparganium eurycarpum* Engelm.		X	4%			OBL	N		Forb	5
Thelypteridaceae	*Thelypteris palustris* Schott		X	4%			OBL	N		Forb	7
Thymelaeaceae	*Thymelaea passerina* (L.) Coss. & Germ.	Hall			8%	50%		E		Forb	
Tiliaceae	*Tilia americana* L.	Hall			i		FACU	N		Tree	5
Typhaceae	*Typha angustifolia* L.			4%	i	i	OBL	B		Forb	
Typhaceae	*Typha latifolia* L.			i	i	10%	OBL	N		Forb	1
Ulmaceae	*Celtis occidentalis* L.		X	4%	12%		FACU	N		Tree	4
Ulmaceae	*Ulmus americana* L.		X	9%	19%	i	FAC	N		Tree	3
Ulmaceae	*Ulmus pumila* L.			9%	27%	40%	UPL	E		Tree	
Urticaceae	*Boehmeria cylindrica* (L.) Sw.	Hall		4%			FACW	N		Forb	6
Urticaceae	*Parietaria pensylvanica* Muhl. ex Willd.		X		8%		FAC	N		Forb	0
Urticaceae	*Urtica dioica* L.		X	4%	4%	i	FAC	B		Forb	1
Verbenaceae	*Phyla cuneifolia* (Torr.) Greene	Hall			i		FAC	N		Forb	4
Verbenaceae	*Phyla lanceolata* (Michx.) Greene		X	39%	35%	30%	FACW	N		Forb	3
Verbenaceae	*Verbena bracteata* Cav. ex Lag. & Rodr.		X	i	4%	10%	FACU	N		Forb	0
Verbenaceae	*Verbena hastata* L.		X	39%	42%	50%	FACW	N		Forb	4
Verbenaceae	*Verbena stricta* Vent.		X	39%	54%	70%	FACU^1^	N		Forb	2
Verbenaceae	*Verbena urticifolia* L.		X	13%	8%		FAC	N		Forb	3
Violaceae	*Viola pedatifida* G. Don	Hall	X				FACU	N		Forb	6
Violaceae	*Viola missouriensis* Greene	Hall		i	i		FACW	N		Forb	4
Violaceae	*Viola sororia* Willd.		X	35%	62%	40%	FAC	N		Forb	3
Vitaceae	*Parthenocissus vitacea* (Knerr) Hitchc.		X	9%	15%	i		N		Vine	4
Vitaceae	*Vitis riparia* Michx.		X	9%	19%	30%	FAC	N		Vine	3
Zygophyllaceae	*Tribulus terrestris* L.		X	4%	i	i		E		Forb	
**Total Species**	**549**	**81**	**320**	**394**	**406**	**338**					

†) n = 23 Mormon Island monitoring plots, ‡) n = 26 Shoemaker Island monitoring plots, ∗) n = 10 Off-Island monitoring plots, 1) Wetland Indicator Status (WIS) estimated via Generalized Linear Models predicting the occurrence of a species based on the presence of multiple common plants with known WISs, 2) Designated a noxious weed in the state of Nebraska. ∼) Nagel and Kolstad (1987) misapplied *S. latifolia* per annotated herbarium specimen (R. Kaul), +) Nagel and Kolstad (1987) misapplied *C. aquatilis* (A. Caven), ˆ) Nagel and Kolstad (1987) likely misapplied *E. dentata* (J. Wiese), >) Kolstad and Nagel (1987) misapplied *D. cladestinum* (J. Wiese). ∞) Specimen was not confirmed by a third-party expert but the species was also recorded by Nagel and Kolstad (1987).

The vast majority of species identified during this study were collected, pressed, and then preserved within the Crane Trust herbarium (Wood River, NE, USA) on acid-free paper, while species representing new county records per Kaul et al. (2012; Hall or Buffalo Cos.) were also sent to the Charles E. Bessey Herbarium at the University of Nebraska–Lincoln (Lincoln, NE, USA). All species identifications were verified by at least two skilled observers and county records were additionally verified by a third-party expert.

### 2.4 Data analysis

We estimated the total number of vascular plant species detected at the Crane Trust's main complex (Mormon Island, Shoemaker Island, off-island sites) over a 40-year period, largely coinciding with its protected status, by integrating results from Nagel and Kolstad (1987; surveys from 1980-1981) as well as verified herbarium records from occasional surveys from 1982-2000 with our survey data from 2015-2020. We calculated the percentage of I&M plots at which each species was detected at all locations from 2015-2020 surveys. We compared our findings to Kaul et al. (2012) to determine if detections represented new records for either Hall or Buffalo County, Nebraska. We also noted, when applicable, the conservation status (Schneider et al., 2018), taxonomic family (Kaul et al., 2012; USDA, 2021a, USDA, 2021b), status as native or exotic as well as growth habitat (USDA, 2021a, USDA, 2021b), wetland indicator status (WIS; Lichvar, 2014), and coefficients of conservatism (CC; Rolfsmeier and Steinauer 2013) for each species detected at the Crane Trust's main complex. We tabulate this data to provide a rich description of the vascular flora detected during our inventory.

We calculated the mean WIS for all plants designated a status at each study location (i.e., Mormon, Shoemaker, and off-island) to provide an indication of how mesic (lower values) or xeric (higher values) each area was (Possible range = 0–5; Lichvar, 2014; Tiner 2016). We increased the number of vascular plants with WIS scores to improve our area WIS estimates using Generalized Linear Models (GLMs) and Pearson's product-moment correlation coefficients (i.e., phi coefficient per binary data; Guilford 1936) employing species presence/absence data from all 59 I&M plots utilizing the “Hmisc” and “stats” packages in R (Nelder and Baker 1972; Harrell 2017; R Core Team 2020). Species were assigned a WIS based on the status of locally common species with known WISs that best predicted their occurrence across plots. Estimates were validated by comparing phi coefficients between plants with unknown WISs and multiple with known statuses (Harrell 2017; R Core Team 2020). If species occurrence was significantly predicted by locally common species of a particular WIS (e.g., FACW) or significantly associated with multiple species of a particular WIS, they were assigned that status (Table 1). To examine the potential influence of hydrological conditions on WIS scores at Mormon Island from 1980-1981 and 2015–2020 we summarized regional precipitation data (NOAA 2021) and river discharge data (USGS 2021) during each survey period as well as the respective periods of record for each database.

We calculated mean CC values (Rolfsmeier and Steinauer 2013) and standard floristic quality index (FQI) values (Swink and Wilhelm 1994; Cretini and Steyer 2011) for Mormon Island from 1980-1981 and 2015–2020, Shoemaker Island from 2015-2020, and off-island habitats from 2015-2020. CC values range from 0-10 for individual species, with plants that receive low values being common ruderal species that can tolerate a wide range of conditions and high values representing rare species that require a relatively narrow set of conditions, and thus often inhabit high quality remnant sites (Swink and Wilhelm 1994). We used species occurrence data per location and time period to calculate the FQI, which provides an effective indicator of site condition that can be used to track changes over time (Lopez and Siobhan Fennessy 2002; Cretini and Steyer 2011).

To determine the direction that each new county record was introduced from, we partitioned Nebraska into several sections, first dividing the state in halves (E-W, N-S) and then into quadrants (SE-SW-NE-NW). We then tallied the total number of counties within each subsection of the state in which new Hall or Buffalo County records documented at the Crane Trust had been previously recorded per Kaul et al. (2012). We used a Pearson's Chi-squared analysis including Bonferroni post-hoc tests following McDonald (2009) to determine if a plant occurrence positively deviated from expected values in one portion of the state. If the chi-squared test was positively significant for only one direction we included that as the predominant direction from which individual species spread. If more than one direction was statistically significant (e.g., E and SE) we selected the predominant direction as the one with the stronger statistical relationship. We then tallied the results to determine the most common direction from which newly established plants came.

We utilized the “simba” package in R to assess the similarity between Mormon Island, Shoemaker Island, and off-island habitats from 2015-2020 as well as Mormon Island from 1980-1981 (Jurasinski and Retzer 2012; R Core Team 2020). We present Jaccard (1912) and Sorensen (1948) similarity indices for pairwise comparisons of species composition across sites using the “sim” function (Jurasinski and Retzer 2012). Secondly, we compare each site and time period using the “mos.f” function which provides a measure of “focal singularity” that serves as an index of similarity between one site/time period (e.g., Mormon Island, 1980–1981) and all others collectively. Finally, we used the “mps” function to calculate Whittaker's *β*-diversity, which essentially measures the diversity across the larger landscape compared to the diversity of each analytical unit within it, in this case Mormon Island, Shoemaker Island, and off-island sites (Whittaker 1960; Jurasinski and Retzer 2012).

## 3 Results

We documented 389 vascular plant species on Mormon Island, 405 species on Shoemaker Island, and 337 species on off-island sites for a total of 520 species detected from 2015 to 2020. Including results from occasional small-scale surveys completed from the mid-1980s to the early 2000s, 394, 406, and 338 species have been detected on Mormon Island, Shoemaker Island, and off-island habitats, respectively (524 species total), since Nagel and Kolstad (1987) documented 320 species on Mormon Island from 1980 to 1981 (Table 1). Nagel and Kolstad (1987) documented 25 species at Mormon Island from 1980 to 1981 that we did not detected at the Crane Trust's main complex (i.e., Mormon Island, Shoemaker Island, and off-island areas) from 2015 to 2020. Conversely, from 2015 to 2020 we documented 225 species at the Crane Trust's main complex not detected from 1980 to 1981 at Mormon Island. This discrepancy is less drastic considering Mormon Island alone, where we documented 126 species from 2015 to 2020 that were not observed from 1980 to 1981 but failed to redetect 57 species captured by the original inventory. Essentially, of the 446 total species detected on Mormon Island from 1980 to 2020, 12.8% were only detected during the initial inventory and 28.3% were only detected during the recent reinventory. In total, 549 vascular plant species were documented at the Crane Trust's main complex between 1980 and 2020 (Table 1). This equates to one unique species for every 11.6 ac (4.7 ha) of habitat surveyed. Our results included 81 county records from Hall and Buffalo counties per Kaul et al. (2012).

**Figure 2:**
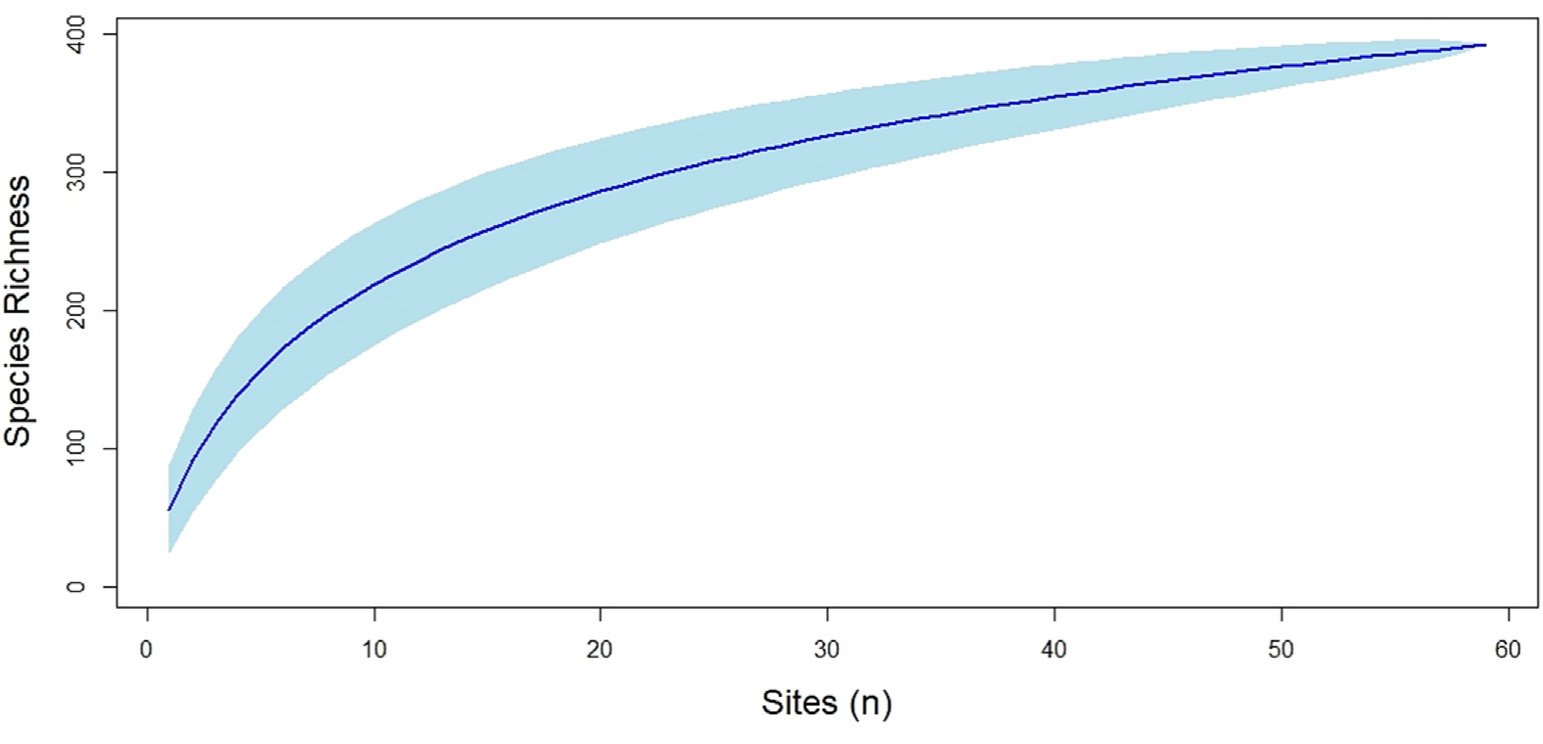
Predicted species richness by number of sites sampled. Species accumulation curve developed using a sample-based rarefaction method where the dark blue line represents the point estimate and the light blue boundary the 95% confidence interval.

During surveys from 2015 to 2020, we detected >70% of vascular plant species on long-term I&M plots at Mormon Island (71.5%, n = 278), Shoemaker Island (73.8%%, n = 299), and off-island (72.1%, n = 244) sites. The rarefaction curve based on our data predicted the number of species added per survey plot would increase steeply across the first 15 plots, more gradually over the next 35, and only incrementally beyond 50 plots (Figure 2). For comparison, the model predicted that sample species richness would increase by an average of 31.33 ± 6.00 (*x̄*±*SE*) species across each of the first 5 plots surveyed but only by an average of 1.68 ± 0.02 across the last 5 (Figure 2).

We documented six species designated as Tier-2 species of concern in the state of Nebraska during 2015–2020 surveys including *Lobelia cardinalis* (cardinal flower)*, Cuscuta gronovii* (swamp dodder)*, Muhlenbergia sylvatica* (woodland muhly)*, Agalinis purpurea* (purple false foxglove)*, Penstemon digitalis* (foxglove beardtongue)*,* and *Penstemon tubaeflorus* (white wand beardtongue)*.* Two of six (*L. cardinalis* and *M. sylvatica*) were previously recorded by Nagel and Kolstad (1987), five of six represented county records per Kaul et al. (2012), and all of them with a designated WIS were categorized as facultative wetland species. Additionally, four of the six were forbs and three of six were in the figwort family. Only one Tier-1 vascular plant species has been documented at the Crane Trust's main complex, *Platanthera praeclara* (western prairie fringed orchid), and we did not detect it during the 2015–2020 reinventory of Mormon Island where it was originally located.

The ten most frequently encountered species across all sites in descending order of abundance were *Panicum virgatum* (switchgrass), *Ambrosia psilostachya* (western ragweed), *Poa pratensis* (Kentucky bluegrass), *Spartina pectinata* (prairie cordgrass)*, Andropogon gerardii* (big bluestem)*, Medicago lupulina* (black medick)*, Sorghastrum nutans* (Indiangrass)*, Agrostis stolonifera* (creeping bentgrass)*, Symphyotrichum ericoides ssp. ericoides* (white heath aster), and *Solidago canadensis* (Canada goldenrod). This includes six grass species (Poaceae), three sunflower species (Asteraceae), and one pea species (Fabaceae), seven species considered native, two species considered exotic, and one species considered both within its current range. Of the ten most frequently encountered species, six were graminoids while four were forbs, and seven are considered facultative upland plants, while two are defined as facultative wetland plants, and one species as facultative. The most frequently encountered tree species were *Ulmus pumila* (Siberian elm), *Populus deltoides* (plains cottonwood), and *Morus alba* (white mulberry). The most widespread vine was *Vitis riparia* (riverbank grape) followed by *Cuscuta glomerata* (rope dodder), and the most common shrub was *Cornus drummondii* (roughleaf dogwood) followed by *Salix exigua* ssp. *interior* (sandbar willow). The most common sedges (*Carex* spp.) were *C. brevior* (shortbeaked sedge), *C. praegracilis* (clustered flatsedge), and *C. pellita* (woolly sedge).

Overall, eleven plant families were represented by at least 10 individual species from our inventory. In descending order, 95 Poaceae (grass), 74 Asteraceae (sunflower), 49 Cyperaceae (sedge), 36 Fabaceae (pea), 19 Lamiaceae (mint), 17 Polygonaceae (smartweed/buckwheat), 16 Scrophulariaceae (figwort), 14 Brassicaceae (mustard), 11 Euphorbiaceae (spurge), 11 Ranunculaceae (buttercup), and 11 Rosaceae (rose) species have been documented at the Crane Trust's main complex to date. By contrast 30 families were represented by just a single species (e.g., Phrymaceae – *Phryma leptostachya* (American lopseed)).

**Figure 3:**
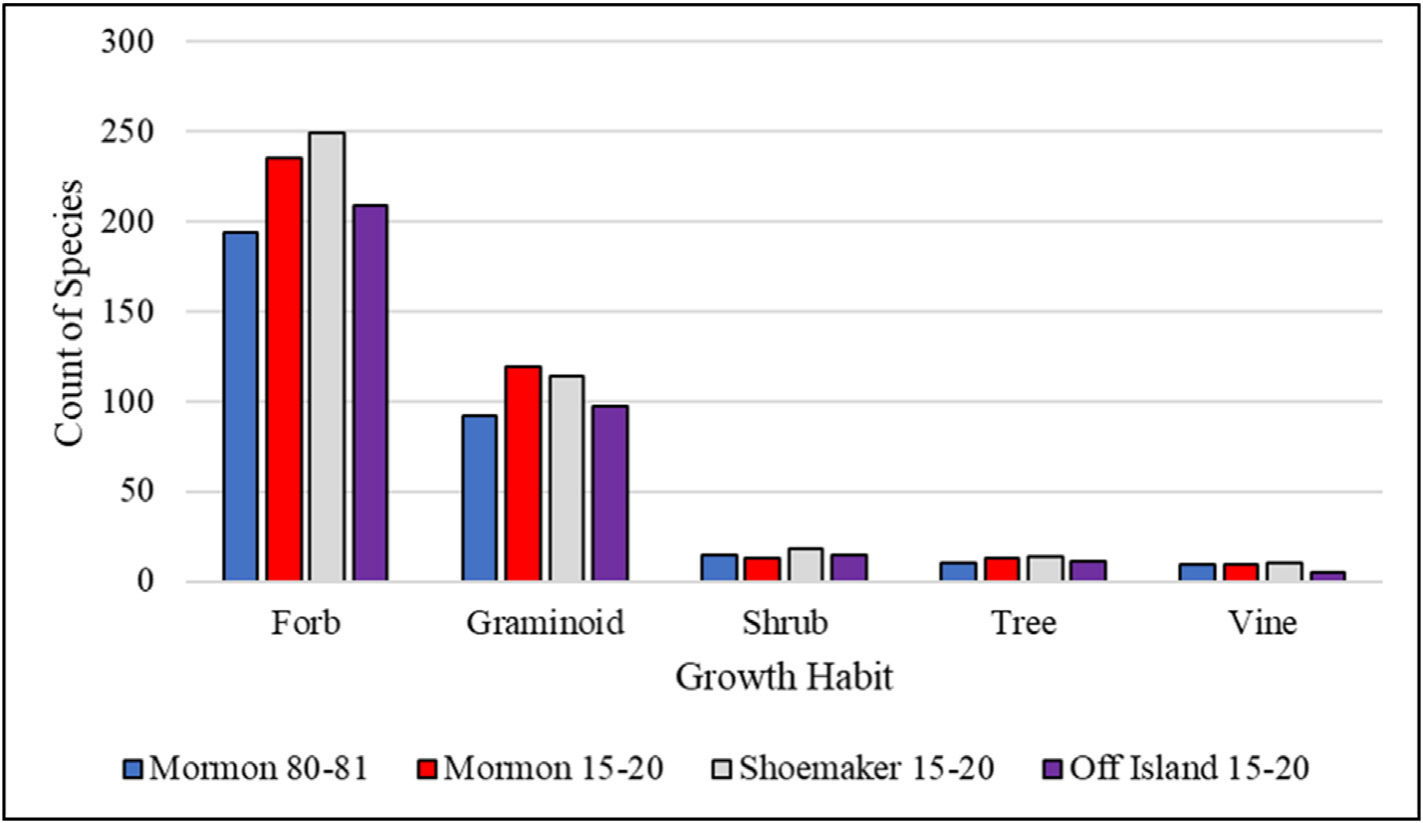
Count of species observed by growth habit during surveys from 2015–2020 at Mormon Island, Shoemaker Island, and off-island habitats as well as from 1980–1981 at Mormon Island.

In total, 345 forb, 152 graminoid, 23 shrub, 16 tree, and 13 vine species have been documented at the Crane Trust's main complex from 1980 to 2020. Comparing results at Mormon Island from 1980-1981 and 2015–2020, recent surveys detected a notably higher number of forb (235 vs. 194), graminoid (119 vs. 92), and tree species (13 vs. 10) compared to historic surveys (1980–1981) as well as fewer shrub (13 vs. 15) and the same number of vine (9) species (Figure 3). Considering 2015–2020 survey results, the highest number of graminoid species was detected on Mormon Island (119), however, Shoemaker Island had the highest number of species in every other growth habit category including forbs (249), shrubs (18), trees (14), and vines (10; Figure 3).

Overall, 76.7% of plants detected on the Crane Trust's main complex from 1980-2020 were native, 20.2% were classified as exotic, and 3.1% were classified as falling under both categories within their current range. Survey results from Mormon Island indicate that 78.8% of the species documented in 1980–1981 were native and results from 2015-2020 indicate that 76.9% of species at Mormon Island, 76.8% at Shoemaker, and 72.7% at off-island sites were native.

**Figure 4:**
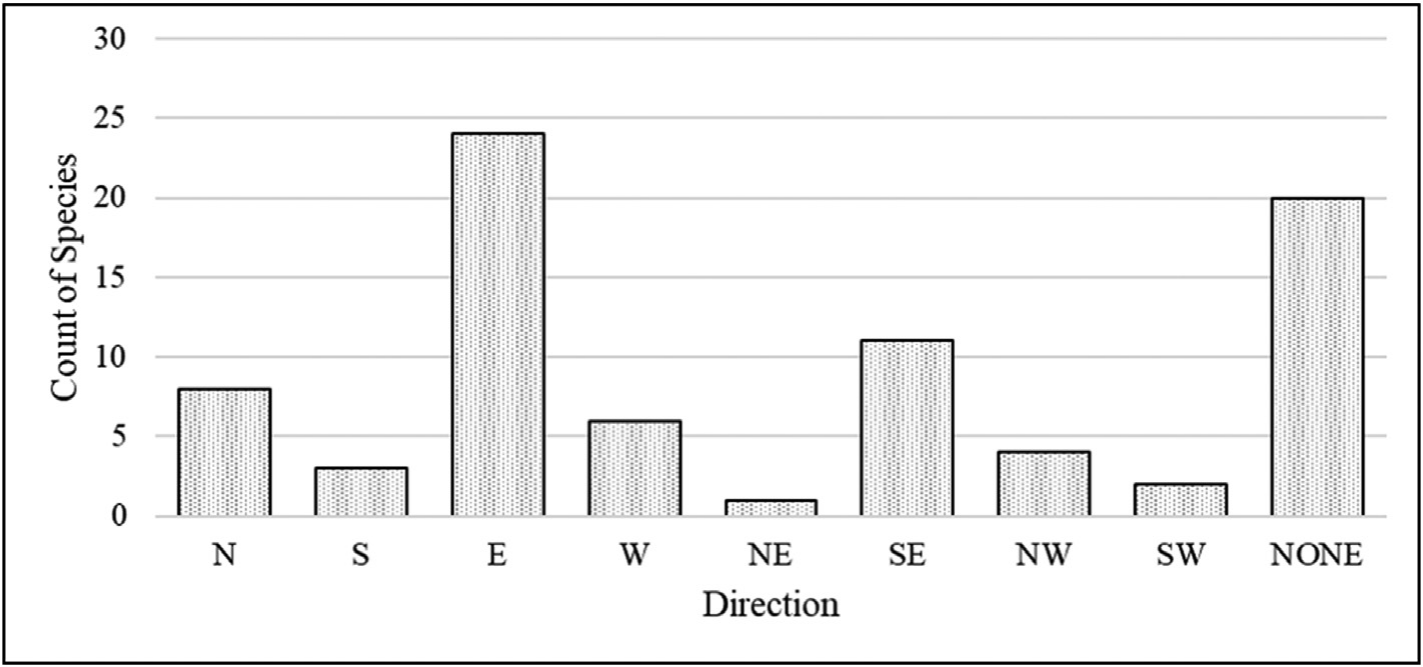
Total number of species that represented distributional records to Hall and Buffalo Counties by direction of origin including North (N), South (S), East (E), West (W), Northeast (NE), Southeast (SE), Northwest (NW), and Southwest (SW). “NONE” indicates that no direction occurred more than expected.

We examined the statewide distributions of 79 out of the 81 county records documented during this study and found that new records did not come equally from all parts of the state (*χ*^2^ = 60.78, *p* = <0.0001). New plants predominantly represented colonization from eastern Nebraska (n = 24; *χ*^*2*^ = 23.28, *p* = <0.0001; Figure 4). Southeastern Nebraska was the second most common source of directional spread but did not differ significantly from expected values (n = 11; Figure 4). However, a significant number of species did not appear to be migrating into our study area from any direction per our analysis (n = 20; *χ*^2^ = 13.09, *p* = 0.0003; Figure 4), and therefore likely represented range gaps partially filled by our study. We estimated that at least 10 county records were introduced via seeding efforts associated with prairie restoration or enhancement along with several additional species novel to the CPRV but previously recorded within Hall and/or Buffalo counties.

Plants detected on Mormon Island from 2015-2020 had a mean WIS of 2.94 compared to a score of 3.06 for those documented in 1980–1981, indicating that the current community may be more hydrophytic and conditions more mesic than 40 years ago. Shoemaker Island represented the driest location based on 2015–2020 data with a mean WIS of 3.13, closely followed by off-island sites (mean WIS = 3.11). Mormon Island had a higher percentage of species defined as obligate wetland (16.5%) and facultative wetland (24.0%) species from 2015-2020 than from 1980-1981 surveys (14.7% and 19.2%, respectively). Considering data across all sites from 2015-2020, Mormon Island had the highest percentage of species defined as obligate and facultative wetland species, while Shoemaker had the highest percent defined as facultative upland species (34.7%), and off-island sites had the highest percentage categorized as facultative (21.7%) and upland species (12.7%; Table 2).

**Table 2:** Percentage of species with an allocated Wetland Indicator Status (WIS) as Obligate Wetland (OBL), Facultative Wetland (FACW), Facultative (FAC), Facultative Upland (FACU), and Upland (UPL) per survey area (Mormon Island, Shoemaker Island, Off-Island) and time period (1980–1981, 2015–2020).

WIS	Mormon Island (1980–1981)	Mormon Island (2015–2020)	Shoemaker Island (2015–2020)	Off-Island (2015–2020)
OBL	14.7%	16.5%	12.9%	12.3%
FACW	19.2%	24.0%	20.3%	21.4%
FAC	22.9%	19.0%	20.3%	21.7%
FACU	32.0%	30.2%	34.7%	31.9%
UPL	11.3%	10.3%	11.9%	12.7%

Based on regional weather data at Minden, Nebraska, from 1893-2021 annual precipitation during our study period (2015–2020) was slightly above average 66.3 ± 6.1 cm (*x̄*±*SE* = 26.1 ± 2.4 in.; *SD* = 15.0 cm, 5.9 in.) compared to the historic record 62.2 ± 1.5 cm (*x̄*±*SE* = 24.5 ± 0.6 in.; *SD* = 17.0 cm, 6.7 in.). Annual precipitation during our study was never more than 1 standard deviation below the mean for the period of record (min. = 46.7 cm; 18.4 in.; 2020) but it did exceed 1 standard deviation above the mean in 2018 (81.5 cm; 32.1 in.) and 2019 (88.4 cm; 34.8 in.). Growing season precipitation totals were also similar between the period of record and our study, averaging 36.3 cm (14.3 in.) cumulative precipitation from May to August from 1893-2021 and 37.3 cm (14.7 in.) from 2015-2020. Annual precipitation was also near or above average during the 1980 (53.8 cm; 21.2 in) and 1981 (83.1 cm; 32.7 in) study period. Growing season (May to August) river discharge was well above average (87.4 ± 3.3 cms; *x̄*±*SE* = 3,087 ± 117 cfs) during our study compared to the period of record for the Platte River at Grand Island, Nebraska, from 1934 to 2021 (*x̄*±*SE* = 45.1 ± 0.7 cms; 1,593 ± 25 cfs). Growing season river discharge was also above average during the 1980–1981 study, but to a lesser degree (*x̄*±*SE* = 63.0 ± 6.0 cms; 2,225 ± 212 cfs).

The mean CC value was 3.69 at Mormon Island based on 2015–2020 data and 3.63 based on 1980–1981 data, indicating a slight positive increase. The mean CC value from 2015-2020 at Shoemaker Island was 3.57 and at off-island sites was 3.58, slightly below averages from Mormon. Standard FQI values for Mormon Island were 57.6 from 1980-1981 and were 63.8 from 2015-2020, again indicating a positive increase. Shoemaker Island (2015–2020) had an FQI value of 63.0, similar to Mormon Island. Off-island sites (2015–2020) had a slightly lower FQI value of 56.0.

Mormon Island from 1980-1981 was more similar to Mormon Island from 2015-2020 on both the Jaccard's Similarity Index (JSI; 0.59) and the Sørensen Similarity Index (SSI; 0.74) than the other recently surveyed areas (Table 3). Mormon Island from 1980-1981 was more similar to Shoemaker Island from 2015-2020 (JSI = 0.54; SSI = 0.70) than off-island sites (JSI = 0.51; SSI = 0.67). However, Mormon Island from 2015-2020 was more similar to Shoemaker Island (JSI = 0.63; SSI = 0.77) and off-island habitats (JSI = 0.61; SSI = 0.75) from 2015-2020 than Mormon Island from 1980-1981 (Table 3).

**Table 3:** Sørensen (bold) and Jaccard (italic) Similarity Indices comparing vegetation at Mormon Island from 2015-2020, Mormon Island from 1980-1981, Shoemaker Island from 2015-2020, and off-island sites from 2015-2020.

Location	Mormon Island (1980–1981)	Mormon Island (2015–2020)	Shoemaker Island (2015–2020)	Off-Island (2015–2020)
Mormon Island (1980–1981)	-	**0.74**	**0.70**	**0.67**
Mormon Island (2015–2020)	*0.59*	-	**0.77**	**0.75**
Shoemaker Island (2015–2020)	*0.54*	*0.63*	-	**0.74**
Off-Island (2015–2020)	*0.51*	*0.61*	*0.59*	-

Sørensen (bold) and Jaccard (italic)" is placed in the figure heading. Bold text corresponds to Sørensen similarity index values and italicized text corresponds to Jaccard similarity index values.

Measures of focal singularity were highest for Mormon Island from 2015-2020 (0.670), followed by off-island 2015–2020 (0.643), Shoemaker Island 2015–2020 (0.640), and Mormon Island 1980–1981 (0.623), indicating that Mormon Island 2015–2020 was most similar to all other sites/periods collectively regarding species presence. Finally, the Whittaker's *β*-diversity score for the Crane Trust's main complex was 1.50 considering the three survey areas.

## 4 Discussion

### 4.1 Habitat classifications

Four of the ten most commonly encountered species represent perennial warm season grasses important to ecosystem function that are indicative of tallgrass prairie habitat (Weaver and Fitzpatrick 1932). Much of the Crane Trust's main complex fits the definition of “lowland tallgrass prairie” described by Kaul et al. (2012) and Rolfsmeier and Steinauer (2010). Slightly more mesic portions of the landscape represent archetypal “northern cordgrass wet prairie” per Rolfsmeier and Steinauer (2010) as well. Though the Crane Trust's main complex contains a significant amount of sedge dominated “wet meadow” and various forms of “marsh,” the dominant vegetation community is the subirrigated lowland tallgrass prairie (Currier 1989; Henszey et al., 2004; Rolfsmeier and Steinauer 2010). However, many transitional forms are present on the landscape, and these represent areas where wetland plants can expand during wet cycles (Currier 1989; Henszey et al., 2004). Mean WISs <3.0 indicate wetland habitat and values 3.0 < 3.2 often represent transitional sites (De Steven 2015; Tiner 2016). Though our results represent data from multiple research plots across islands and non-island locations, they indicate a majority of species on Mormon Island are indicative wetland landcover, and that the other locations contain a significant number of sites with wetland plant species that taken together average a WIS associated with transitional habitats.

Our results generally correspond to McKee (2006), which indicated that hydric soils comprised about 35% of herbaceous habitats in the CPRV, and that wet meadow features are usually embedded within larger tracts of lowland tallgrass prairie. However, wetland plant communities and associated fauna can expand and contract spatially over subsequent wet and dry cycles, and several species may be present under only certain hydrological conditions (Currier 1984, 1989; Currier and Henszey 1996; Henszey et al., 2004, Davis et al., 2006). Precipitation and river discharge patterns have a substantial impact on subirrigated lowland tallgrass prairie and wet meadow ecosystems (Wesche et al., 1994; Davis et al., 2006; Brinley Buckley et al., 2021a). Our finding that Mormon Island was wetter per the vegetation WIS score from 2015-2020 than from 1980-1981 was likely reflective of the relatively wet hydrological conditions present during a significant portion of our study.

Whittaker's *β*-diversity measure suggests that the Crane Trust's main complex hosts 1.5 species for every one identified at each location (Whittaker 1960), or as Jost (2007) suggests it has 1.5 distinct communities. This result indicates that although there is significant overlap between each island and the off-island habitat, there are also distinctive features at each. For instance, only Shoemaker Island contains *Shepherdia argentea* (silver buffaloberry) and the silver buffaloberry shrubland community is considered “vulnerable to extirpation” within Nebraska (S2 rank; Rolfsmeier and Steinauer 2010). Similarly, Mormon Island contains a wider range of wetland habitats, with several grading between wet meadow and shallow marsh that contain unique wetland plants such as *Carex stipata* (awl-fruited sedge) and *Onoclea sensibilis* (sensitive fern), which previous to our study was thought to be extirpated from the CPRV (Kaul et al., 2012). Finally, off-island sites had more early-successional and restored habitats that tended to include vascular plants valuable to pollinators that were generally not present on Mormon or Shoemaker Islands such as *Astragalus canadensis* (Canadian milkvetch) and *Penstemon digitalis*.

### 4.2 Restoration influences

The fact that Mormon Island from 2015-2020 is more similar to Shoemaker Island (SSI = 0.77) and off-island sites (SSI = 0.75) from the same period than Mormon Island from 1980-1981 (SSI = 0.74) indicates that changes in the vegetation community at the Crane Trust over the last 40 years are likely pervasive and to some extent homogenizing. In other words, the fact we observed more similarity across space than time suggests that changes in the vegetation at Mormon Island probably reflect widespread community shifts observed throughout the study area. It is also notable that Nagel and Kolstad (1987) demonstrated an SSI of 0.75 between Mormon Island and Rowe Sanctuary from 1980-1981, which is also slightly higher than the SSI we observed between Mormon Island from 1980-1981 and from 2015-2020 (Table 3). Drivers of change likely include continued restoration work with similar practices applied throughout, such as the application of seed mixes dominated by easy to harvest species, especially considering older restorations (Pfeiffer 1999; Whitney 1999; Larson et al., 2021). As Pfeiffer (1999) notes early cropland restoration efforts often contained only 3 to 6 species, whereas more recent restorations included >100 species. Data indicates a relatively steady increase in lowland grassland within the floodplain of the CPRV since the 1980s associated with restoration efforts by conservation organizations (Crane Trust, Audubon, The Natural Conservancy; Krapu et al., 2014; Caven et al., 2019b). Krapu et al. (2014) documented a 31.7% increase in “lowland grasses” between Chapman and Lexington, Nebraska, within 5.6 km of Platte River from 1982 to 1998. Similarly, Caven et al., 2019a, Caven et al., 2019b documented a relatively modest increase in “meadow-prairie” landcover from 1998 to 2016 within 800 m of the main channel of the Platte River between Chapman and Overton, Nebraska (*x̄* = +2.8% per river segment, range = -0.9% to +12.1%, n = 11). However, in both cases these gains were uneven and concentrated around lands owned and managed by conservation organizations (Krapu et al., 2014; Caven et al., 2019b).

Expanded areas of restored tallgrass prairie provide an explanation for how the vegetation community has increased in floristic quality while paradoxically becoming more homogenous over the last 40 years of restoration efforts (Pfeiffer 1999; Whitney 1999; Spyreas 2019). Our results demonstrate an increase in species richness at Mormon Island from the 1980–1981 to the 2015–2020 study period and indicate that many species were likely added to the landscape through restoration (Pfeiffer 1999; Whitney 1999, Table 1). Restorations have the ability to buffer high-quality remnant sites from the most problematic exotic species invasions (Gerla et al., 2012; Rowe, 2013) but can themselves introduce novel species, albeit those that tend to be of higher floristic quality and generally native to the larger ecoregion (Pfeiffer 1999; Whitney 1999; Trowbridge et al., 2017; Egawa 2017; Spyreas 2019; Larson et al., 2021). As Spyreas (2019) notes, many restoration plantings include a small regional subset of species with relatively high CC values that are “over-promoted” because they are comparatively easy to harvest, establish, or purchase. Therefore, restorations often replace distinct local communities that have been lost with more general regional communities, which ultimately contributes to “biological homogenization” while improving floristic quality (Russo et al., 2013; Williams et al., 2018; Spyreas 2019). Some “native” species introduced to Mormon Island were relatively common in Hall County, Nebraska, but generally absent from lowland grasslands within the alluvial CPRV (Nagel 1981; Nagel and Kolstad 1987; Kaul et al., 2012). Illustrative examples include *Amorpha canescens* (leadplant) and *Helianthus pauciflorus* (stiff sunflower), which are generally widespread in drier upland prairie sites, such as nearby Sandhills prairies (aeolian soils; Rolfsmeier and Steinauer 2010; Kaul et al., 2012). However, some native species introduced to Mormon Island, Shoemaker Island, and/or off-islands habitats represent relatively rare species, at least regionally.

We estimated ≥10 species native to the central Great Plains but novel to the CPRV as well as Hall County, Nebraska, were introduced to the Crane Trust through restoration efforts over the last four decades (Kaul et al., 2012). These included *Penstemon digitalis* and *Penstemon tubaeflorus*, which both represent tier-2 species of conservation concern per the Nebraska Game and Parks Commission and have CC values of 6 (Rolfsmeier and Steinauer 2013; Schneider et al., 2018). Novel reintroduction may provide a useful conservation strategy for vascular plant species of concern (Wendelberger et al., 2008) and could provide additional floral resources and therefore resilience to declining pollinator populations (Russo et al., 2013; Williams et al., 2018). However, caution should be taken to ensure novel introductions do not compete with or displace local analogs (Gustafson et al., 2004; Rowe 2010; Williams et al., 2018; Larson et al., 2021). Using local ecotype seed for restoration efforts, which is optimally adapted to local environmental conditions, represents a best practice that should *prima facie* limit the scope of novel introductions if rigorously applied to future restorations (Rowe 2010; Larson et al., 2021).

### 4.3 Invasive species influences

The continued expansion of common exotic-invasive species (e.g., *Bromus inermis* – smooth brome, *Lythrum salicaria* – purple loosestrife) and colonization by novel exotic-invasive species (e.g., *Leonurus cardiaca* ssp. *cardiaca* – motherwort*, Iris pseudacorus –* yellow flag iris) also appears to be a notable source of homogenization across sites (Sharma et al., 2005; Seabloom et al., 2006). Our data indicates that about 33% of new county records were exotic species. Nagel and Kolstad (1987) only incidentally documented *Lythrum salicaria* off-transect but we documented the species at 39% of I&M plots at Mormon Island, 27% at Shoemaker Island, and 20% at off-island habitats. Our results provide the percentage of plots at which each species was detected per area while Nagel and Kolstad (1987) presented the average cover of species per quadrat sample. Nonetheless, the increasing number and cover of exotic-invasive species is clearly described by comparing our respective findings. For example, *Phragmites australis* was not detected at Mormon Island from 1980 to 1981 (Nagel and Kolstad 1987) but it was detected on 9% of I&M plots at Mormon Island, 4% at Shoemaker Island, and 20% at off-island habitats from 2015 to 2020. However, the extent of recent *P. australis* invasion in the CPRV is not clearly expressed by the abundance estimates presented herein as survey data were collected mostly from permanent islands and bank habitats. *P. australis* primarily colonizes dry portions of the active channel bed (i.e., “temporary sandbars”) as well as bare ground within off-channel wetlands, which has resulted in reduced channel capacity and functionality in the CPRV during growing seasons with consistently low flows (Rapp et al., 2012; Galatowitsch et al., 2016). Similarly, the most widespread tree species overall on Crane Trust properties from 2015 to 2020 was the exotic-invasive *Ulmus pumila*, which was not detected by Nagel and Kolstad (1987) from 1980 to 1981.

*U. pumila* was comparatively widespread because seedlings and saplings were regularly encountered invading predominantly herbaceous habitats. *U. pumila* was also the most widespread tree species because the Crane Trust focused on clearing riparian woodland dominated by *Populus deltoides* and replacing it with herbaceous vegetation for the benefit of cranes over the last 40 years (*Grus* spp.; Currier et al., 1985; Currier 1991). *P. deltoides* remains the second most widespread tree overall (13.6% of all I&M plots) but is not dominant across island or off-island areas. Tree species occurrence varied markedly between islands. *Fraxinus pennsylvanica* (green ash), *M. alba*, and *P. deltoides* were the most widespread tree species on Mormon Island, *U. pumila*, *Juniperus virginiana* (eastern redcedar), and *M. alba* were most abundant on Shoemaker Island, and *U. pumila*, *P. deltoides*, and *M. alba* were most common on off-island habitats, respectively. Continued restoration work for the benefit of cranes along with exotic-invasive species colonization have likely together altered the projected course of natural woodland succession proposed by Currier (1982) in this reach of the CPRV and *U. pumila* will likely continue to become more dominant in the absence of effective control efforts.

Our data also demonstrates an increase in the distribution of native species that can invade and modify remnant herbaceous habitats as well. For instance, *J. virginiana* invasion can alter soil chemistry and site hydrology, increase wildfire risks, reduce herbaceous biomass production, and decrease biodiversity in grasslands (Briggs et al., 2002; Horncastle et al., 2005; McKinley et al., 2008; Twidwell et al., 2013; Zou et al., 2018). Currier (1982) indicates that *J. virginiana* did not become measurably established on Mormon Island until the 1950s. Accordingly, Nagel and Kolstad (1987) did not detect *J. virginiana* along systematic transects from 1980-1981 but did note the species' presence via supplemental collections. We detected *J. virginiana* on 27% of I&M plots at Shoemaker Island, 4% at Mormon Island, and incidentally at off-island sites. *Phalaris arundinacea* (Reed canary grass) represents another “native-invasive” that can reduce diversity across taxa in wetland habitats, though research indicates genotypes introduced from Europe may be contributing to the species’ aggressiveness and dominance (Lavergne and Molofsky 2004; Schooler et al., 2006). Nagel and Kolstad (1987) recorded only trace cover of *P. arundinacea* along transects at Mormon Island from 1980-1981. However, we recorded *P. arundinacea* on 50% of I&M plots at Shoemaker Island, 39% at Mormon Island, and 30% at off-island areas. Our findings highlight the growing influence of invasive species at the landscape level despite management and restoration efforts intended to limit their distributions and abundances, which is broadly reflective of trends in the Great Plains (Symstad and Leis 2017; Fogarty et al., 2020; Gaskin et al., 2021). Efforts should be made to monitor emerging invasive species threats (See http://neinvasives.com/plants) and improve invasive species control efforts (e.g., apply a 3–5-year fire return interval, targeted grazing, etc.) to maintain herbaceous habitat quality in the CPRV (Buehring et al., 1971; Herrick et al., 2009; Ratajczak et al., 2014; Alstad et al., 2016).

### 4.4 Range expansions

Our analysis suggested that the primary direction from which vascular plants spread into Hall County, Nebraska, was from the east and to a lesser extent the southeast. Though some of the county records regarding native species from eastern and southeastern Nebraska likely represent introductions through restoration (e.g., *Penstemon digitalis*, *Silphium integrifolium* var. *laeve*), others represent inconspicuous species that are challenging to collected, not generally included in seed mixes, and therefore potentially represent climate-related westward or northwestward expansion. Illustrative examples include, *Ammannia coccinea* (valley redstem), *Erechtites hieraciifolius* (American burnweed), *Hackelia virginiana* (beggarslice), *Carex blanda* (eastern woodland sedge), *Juncus tenuis* (poverty rush), *Muhlenbergia schreberi* (nimblewill), and *Corydalis micrantha* ssp. *micrantha* (smallflower fumewort). Recent research in south central Nebraska has documented range expansions northward and westward for several animal species, often along the Platte River corridor, but less research has addressed vascular plant range shifts regionally (Thompson and Finck 2013; Geluso et al., 2014; Carlson and Geluso 2018; Forrester et al., 2019). Plant hardiness zones have shifted northward and westward along Nebraska's elevational gradient (Daly et al., 2012). As growing season length and minimum temperatures increase throughout the state it is possible that future climate-related range expansions will include westerly as well as northerly movements regionally for species not limited by arid western conditions.

### 4.5 Floristic quality

It is notable that Mormon Island is the largest, most contiguous, and experiences the least frequent and lowest intensity anthropogenic disturbances of all our study locations. The fact that it scores highest on floristic quality measures fits with a number of ecological theories including the theory of island biogeography (MacArthur and Wilson 1967; Lindgren and Cousins 2017). This theory posits that larger and more connected habitats will have a higher natural species immigration rate and lower extinction rate and therefore will support higher native species richness, which is an important component of the FQI calculation (MacArthur and Wilson 1967; Wilhelm and Masters 1995; Lindgren and Cousins 2017). As Wilhelm and Masters (1995) note, FQI values ≥ 35 are indicative of a relatively “natural” floral communities, and values ≥ 45 indicate that a site is likely a remnant in natural condition. By these standards all three areas assessed at the Crane Trust from 2015-2020 had floral communities indicative of remnant natural areas, as did Mormon Island from 1980-1981 (range = 56.0–63.8; Wilhelm and Masters 1995).

Mormon Island, Shoemaker Island, and off-island areas displayed higher FQI values than most other herbaceous habitats assessed in the region (Kottas 2001; Jog et al., 2006; Miller 2008; Rothenberger et al. 2010, 2014; Hastings and Rothenberger 2013; Flynn and Rothenberger 2014; Soper, 2018). For instance, of 104 grasslands assessed by Jog et al. (2006) in northeastern Kansas, the highest FQI observed was 41.0 in a warm season hay meadow. Similarly, all three sites assessed by Hastings and Rothenberger (2013) along Nebraska's Republican River had FQI values of <25.1. Additionally, all sites evaluated by Flynn and Rothenberger (2014) along the Loup River Valley, Nebraska, had FQI values < 38.0.

Only a small number of published reports from Nebraska detail FQI values similar to those at Mormon Island and adjacent habitats (Kottas 2001; Rothenberger et al. 2010, 2014). For instance, Rothenberger et al. (2014) described a 40-ha (99 ac) wet meadow with an FQI of 52.4 along the South Loup River. Kottas (2001) described two remnant tallgrass prairies near Lincoln, Nebraska, with similarly high standard FQI values. The 247 ha (610 ac) Spring Creek Prairie had an FQI value of 53.0 and the 97 ha (240 ac) Nine-Mile Prairie had an FQI value of 63.7, equal to that of Mormon Island. Our search of the Nebraska botanical literature found only a few sites that exceeded Mormon Island in FQI. For instance, the relatively small (16.2 ha; 40 ac) and subirrigated Thomsen Meadow along the Middle Loup River near Rockville, Nebraska, supported an impressive 281 species and had an FQI of 64.4 (Rothenberger et al., 2010). Our results indicate that FQI values at Mormon Island and adjacent conservation areas rank among the highest assessed in Nebraska in recent years despite growing threats from invasive species (Kottas 2001; Rothenberger et al. 2010, 2014). To preserve the quality of Mormon Island it will be essential to effectively control invasive species as well as garner protections for neighboring privately owned prairie and meadow remnants at risk for development (MacArthur and Wilson 1967; Sharma et al., 2005; Rowe, 2013; Twidwell et al., 2013; Alstad et al., 2016; Lindgren and Cousins 2017).

### 4.6 *Platanthera praeclara* disappearance

Though our results indicate we have retained the majority of vascular plant species documented at Mormon Island by Nagel and Kolstad (1987) we did not detect the Federally Threatened *Platanthera praeclara* during our 2015–2020 surveys, which has not been detected at Mormon Island since 2000 (USFWS 1989; Caven 2022). However, research indicates that the WPFO can persist underground as a rhizome or manifest above ground as just 1–3 leaves during the growing season (Siege and King 1995; Sather and Anderson 2012; Smith 2012). Additionally, some research indicates that seed may remain viable in the soil for an extended period of time (Hof et al., 1999). Though WPFO appears to tolerate a range of hydrological conditions and management practices, it may require a narrow set of circumstances to flower *en masse* (Currier 1984; Sheviak and Bowles 1986; Bjugstad-Porter 1993; Bleho, 2015). Moreover, recent detections of known WPFO pollinators within the CPRV suggests that pollinator decline was not likely a key reason for the WPFO's apparent disappearance from Mormon Island (Cuthrell 1994; Westwood and Borkowsky 2004; Travers et al., 2011; Lotts et al., 2021). The population could have been negatively impacted by inbreeding depression associated with geographic isolation or potentially by herbicide drift related to exotic-invasive species control efforts in the area (Kraemer and Alsum 2006; Ross and Travers 2016). However, based on the general condition of Mormon Island, which has retained a number of plants associated with WPFO occurrence, we suggest that there is still a chance the species persists locally and simply went undetected via our survey methods (Currier 1982; Siege and King 1995; Sather and Anderson 2012). Future research efforts should continue to monitor sites of historic occurrence for WPFO emergence and flowering (Caven 2022).

### 4.7 Study considerations

Our results indicate that our I&M program effectively documented the majority of species present on the landscape (>70%). Our documentation of more species than Nagel and Kolstad (1987), including several native species that were likely present at the time of their surveys, further indicates that our I&M approach effectively captured a large proportion of the floral diversity present on the landscape. This perspective is further bolstered by the observation that our study may have filled a number of range gaps at the county-level and that the number of new species added per survey plot increased only marginally beyond a sample of 50. Additionally, the 5-year duration of our systematic study (6 years including incidental detections) and the relatively consistent moisture in multiple years likely assisted our team in detecting several less-common species, including those present only or predominantly in temporary wetlands. One limitation of our analysis is that we used a different and arguably more sophisticated sampling method than the original inventory to set the stage for future long-term monitoring efforts. This, in addition to our comparatively large sampling effort, likely resulted in the detection of some species missed by Nagel and Kolstad (1987). Therefore, observed increases in species richness may reflect improved methods as well as changes within the vegetation community over time to some unknown degree.

Our analysis was conducted at the “landscape-scale” (*sensu* Ingegnoli 2013, “ecodistrict-scale” *sensu* Klijn and Udo de Haes 1994) using species presence/absence data from vegetation inventory and monitoring plots located across respective island and non-island habitats. The use of presence/absence data is a relatively common practice for comparing vegetation communities as it performs similarly to or better than abundance data regarding various analytical approaches at broader scales (Otypková and Chytry 2006; Bastow Wilson 2012). However, patterns of vegetation composition can vary significantly across spatial scales (e.g., Gross et al., 2000; Eiserhardt et al., 2011) and abundance estimates can increase the sensitivity of data to community shifts (e.g., Mason and French 2008). Future research should investigate the impacts of restoration practices, exotic species invasions, and hydrological fluctuations on the vegetation community at Mormon Island and other remnant sites in the CPRV at additional spatial scales (e.g., ecotope-level, ecoregion-level) and data resolutions (e.g., percent cover, biomass, etc.).

## 5 Conclusions

A total of 549 species of vascular plants were detected on the surveyed properties owned and managed by the Crane Trust from 1980 to 2020, equating to about 1 unique plant species per 4.7 ha of conserved land. The most abundant plant families were Poaceae, Asteraceae, Cyperaceae, Fabaceae, and Lamiaceae. Species composition reflected the landscape's designation as “lowland tallgrass prairie” with embedded wetlands such as “wet meadows” (Weaver and Fitzpatrick 1932; Rolfsmeier and Steinauer 2010; Kaul et al., 2012). The transitional nature of the study area was further demonstrated by the WIS scores for all three study sites, which ranged from 2.94 to 3.13, where 3.0 represents the threshold distinguishing wetland from upland habitats (De Steven 2015; Tiner 2016). Mormon Island during both survey periods (1980–1981, 2015–2020) as well as Shoemaker Island and off-island habitats from 2015-2020 demonstrated FQI scores associated with remnant communities in natural condition (range = 56.0–63.8; Wilhelm and Masters 1995). In particular, 2015–2020 FQI values for Mormon (63.8) and Shoemaker (63.0) Islands closely mirrored those from the highest quality herbaceous sites assessed in Nebraska per the published literature such as Nine-Mile Prairie (63.7; Kottas 2001) and Thomsen Meadow (64.4; Rothenberger et al., 2010). These findings suggest that in addition to being considered a highly important migratory bird area, Mormon Island should be recognized as a significant botanical preserve at the state level (Currier 1982; Currier et al., 1985; NRC 2005).

Species richness and floristic quality increased while the mean wetland indicator score decreased (i.e., currently more mesic) since the initial inventory of Mormon Island, which are positive indicators of ecosystem and vegetation community health (Henszey et al., 2004; Davis et al., 2006; Brinley Buckley et al., 2021a). These results likely reflect recent efforts to maintain spring base flows in the Platte River for Whooping Cranes and other species of concern, in addition to wetter than average conditions throughout the study period (NRC 2005). However, our results also indicate the number and spatial distribution of invasive species has increased over the last 40 years, which echoes findings from Alstad et al. (2016) that annual rates of species colonization and extirpation have increased. The ecosystem has retained a significant portion of its original components since 1980–1981 and has added additional native species through restorations, which may increase pollinator resources and help conserve individual plant species of concern (e.g., *P. tubaeflorus*; Russo et al., 2013; Williams et al., 2018). However, additions of locally novel but regionally native species could potentially displace local analogs and caution should be taken regarding future restorations (Gustafson et al., 2004; Larson et al., 2021). Using the most local seed sources available for restorations may help reduce unintended introductions and thereby limit homogenizing influences (Gustafson et al., 2004; Spyreas 2019; Larson et al., 2021). To protect the ecological integrity of Mormon Island and the adjacent landscape it will be important to control invasive species, likely using a multitude of approaches, and to conserve unprotected remnant prairies nearby to support population connectivity for native species (MacArthur and Wilson 1967; Buehring et al., 1971; Ratajczak et al., 2014; Galatowitsch et al., 2016). Restoring additional tracts between and surrounding Mormon Island, Shoemaker Island, and off-island sites with local ecotype seed sources may be a useful approach to buffering remnant tracts from increasing colonization by novel invasive species (Rowe, 2013; Egawa 2017). Future research should continue to examine species community trends at remnant sites on different spatial scales and time frequencies to identify emerging problems so targeted management efforts can be made to mitigate them.

## Declarations

### Author contribution statement

A.J. Caven: Conceived and designed the experiments; Performed the experiments; Analyzed and interpreted the data; Contributed reagents, materials, analysis tools or data; Wrote the paper.

J.D. Wiese: Performed the experiments; Analyzed and interpreted the data; Contributed reagents, materials, analysis tools or data; Wrote the paper.

### Funding statement

This research did not receive any specific grant from funding agencies in the public, commercial, or not-for-profit sectors. Funding for the project was provided directly by the Platte River Whooping Crane Maintenance Trust, Inc. (Doing business as "The Crane Trust").

### Data availability statement

Table 1 of the article includes all necessary information to replicate the vast majority of statistical analyses.

### Declaration of interests statement

The authors declare no conflict of interest.

### Additional information

No additional information is available for this paper.
